# Significance of Vitamins A and E in Cancer Progression and Prevention

**DOI:** 10.3390/ijms262311588

**Published:** 2025-11-29

**Authors:** Jesse T. Kupfer, Noah Boekweg, Hailiang Zheng, John Puckett, Kota V. Ramana

**Affiliations:** Department of Biomedical Sciences, Noorda College of Osteopathic Medicine, Provo, UT 84606, USA

**Keywords:** fat-soluble vitamins, retinol, cancer, tocopherol, oxidative stress

## Abstract

Fat-soluble vitamins, such as vitamins A and E, are essential micronutrients generally found in fruits, nuts, oils, and vegetables. These vitamins have better absorption and retention in the body when compared to water-soluble vitamins. They also play a significant role in cellular metabolism and the pathophysiology of human health and disease. Further, acting as coenzymes in several biochemical pathways, these vitamins also play a crucial role in immune regulation, vision, and oxidative stress responses. Further, these vitamins have emerged as potential preventive and therapeutic strategies for a wide range of diseases. Recently, vitamins A and E have been shown to exert beneficial effects against various cancers. Further, these vitamins are actively involved in cancer progression or prevention by regulating oxidative, immune, and inflammatory responses, as well as epigenetic processes. This narrative review discusses how recent preclinical and clinical studies have identified multiple pathways through which these vitamins impact cancer prevention and therapy. Furthermore, it also analyzes the potential of vitamins A and E in cancer management and advocates for continued research to unlock their therapeutic potential.

## 1. Introduction

Fat-soluble vitamins such as A, D, E, and K are essential micronutrients that significantly regulate the metabolic and signaling pathways involved in the pathophysiology of human health and disease [1]. Further, when compared to water-soluble vitamins, fat-soluble vitamins are stored in the adipose and liver tissues. They have unique absorption mechanisms that depend on dietary fats and bile acids [2]. Fat-soluble vitamins influence a wide range of physiological processes, such as regulation of immune function and metabolism, cell division and differentiation, bone, and skin health, and, most importantly, antioxidant defense [3,4,5]. Further, the storage capacity of these vitamins enables the body to utilize them over extended periods. Therefore, excessive intake of vitamins could lead to unwanted complications.

In addition, the regulatory functions of fat-soluble vitamins are mediated through various molecular and cellular pathways in the body. For example, vitamins can act as cofactors for enzymatic reactions, modulate the activity of nuclear receptors, and protect cellular components from oxidative stress damage. These functions are critical to maintaining cellular homeostasis and maintaining the redox and immune balance in the body. Fat-soluble vitamins are also involved in various physiological processes, such as gene regulation, oxidative, and immune responses, which contribute to the pathophysiology of various human diseases, including cancer [6,7,8]. Recent studies also suggested that fat-soluble vitamins influence cell proliferation, invasion, migration, inflammation, and angiogenesis, which are essential for cancer progression and spread [9,10,11].

Cancer progression requires a complex interaction of genetic, environmental, and lifestyle factors, including nutrient intake. Further, recent studies suggest the potential role of vitamins in influencing cancer risk and progression [9,10,11]. Some of the studies have also shown the use of vitamin supplementation as a preventive and therapeutic strategy [12,13,14]. However, the outcomes of these studies have often been inconsistent and restricted to preclinical cell culture and animal studies. Although a few clinical and epidemiological studies suggest their beneficial effects, additional studies are needed to understand the significance of fat-soluble vitamins in controlling human diseases, especially cancer.

Moreover, since cancer is a complicated disease, the relationship between fat-soluble vitamins and cancer is influenced by numerous factors. These factors include dietary habits, genetic predispositions, environmental exposure, and lifestyle habits. A few studies also suggest that vitamin deficiencies could alter normal cellular processes and increase the risk of cancer development or progression [15,16,17]. On the other hand, adequate intake of vitamins could be associated with a reduced risk of cancer and improved outcomes in some patient populations [18,19]. Therefore, understanding the importance of a well-balanced diet is critical for maintaining a healthy life.

Thus, recent studies suggest that fat-soluble vitamins are crucial in maintaining health and preventing disease, including cancer. In this review article, we specifically discussed how fat-soluble vitamins, such as vitamins A and E, could modulate key biological processes that play a potential role in cancer progression, prevention, and management (Table 1). Further, we aimed to provide a comprehensive understanding of how these essential nutrients can influence cancer outcomes. We conducted a search on PubMed and Google Scholar to find articles published in the last 10 years or so, using keywords such as vitamin A, retinal, carotenoids, vitamin E, tocopherol, tocotrienol, and various cancer types, including leukemia, skin, breast, ovarian, lung, and colon cancers. Further, in this article, we included research articles, comprehensive narrative reviews, systematic reviews, and clinical and preclinical studies to understand the significance of these two vitamins in cancer. We did not include studies on water-soluble vitamins such as A, B, and C or other fat-soluble vitamins such as D and K. Please refer to our recently published article on understanding the role of water-soluble vitamins in cancer progression and prevention [20]. The role of vitamin D in cancer has been extensively investigated, as has the role of vitamin K. Due to the length of this manuscript, we omitted these vitamins in this article, and we will be working on another article specifically directed towards vitamins D and K. This narrative review article discusses the role of vitamins A and E in cancer biology and explores their potential in cancer prevention and management strategies.

## 2. Vitamin A in Cancer Prevention and Treatment

Vitamin A is a group of fat-soluble micronutrients. They are essential for vision, reproduction, growth, and immune function [21]. Generally, vitamin A includes preformed vitamin A (all-trans-retinol (ATRA)), its esters, and provitamin A (carotenoids). Retinyl esters and beta-carotene are metabolized to form retinol, which can be further oxidized to retinal and retinoic acid, which are active vitamin A metabolites [22]. The primary dietary source of vitamin A is animal tissues in their retinyl ester storage form. Other dietary sources include liver, kidney, oil, eel, milk, butter, and egg yolks [23]. Provitamin A is obtained from plant foods such as carrots and spinach and is responsible for the yellow, orange, and red pigments in fruits and vegetables [23].

Preformed vitamin A retinyl esters are absorbed from the diet and hydrolyzed in the intestine into retinol and free fatty acids. Further, provitamin A is either cleaved into retinal and then reduced to retinol, or it is absorbed in its provitamin form. Retinyl esters are almost entirely absorbed by intestinal epithelial cells (70–90%), whereas carotenoids are much less bioavailable (3%) [24]. Regardless of the route of retinol formation, it is transported into the enterocyte, re-esterified, and then transported to the liver via chylomicrons to be stored and released when needed. Upon release from the liver, retinol is transported to extrahepatic tissues by a retinol-binding protein–transthyretin complex. Once transported into the target cell, an intracellular retinol-binding protein (RBP) carries retinol to the nucleus, where it exerts transcriptional control over various genes [25].

Retinal is the aldehyde form of vitamin A generated via the oxidation of retinol. Its necessity in the visual cycle has been well studied [26]. Rhodopsin is a light-sensitive retinal pigment that consists of retinal bound to the protein opsin. When exposed to light, photochemical isomerization of rhodopsin initiates a signal transduction pathway that triggers a nerve impulse to the brain, facilitating the visual cycle [26,27]. Night blindness (nyctalopia) is one of the earliest signs of vitamin A deficiency [28]. Chronic vitamin A deficiency can led to irreversible loss of visual cells, xeropthalmia, and dryness of the conjunctiva and cornea, eventually leading to blindness [28,29].

Further, various studies have shown the protective effects of vitamin A, especially beta-carotene, as a potent antioxidant and anti-inflammatory [30]. The conjugated double bonds present in the carotenoids absorb electrons and neutralize reactive oxygen species [31,32]. Additionally, vitamin A could also play a key role in adaptive immunity, functioning as a cofactor in differentiating T-cells through the induction of IL-2 [33]. It also regulates NK cell function and IFN-g production [34]. Specifically, retinol has been shown to act as a cofactor for B-lymphocyte growth and T-lymphocyte activation [35]. Therefore, vitamin A deficiency can diminish the immune response to pathogens, as well as increase the severity and length of disease. Moreover, vitamin A plays a role in organ development in the embryo, as well as the formation of the reproductive system in females and males [36].

Recent studies have shown that vitamin A is associated with the development and mitigation of several cancers, including liver, lung, breast, cervical, skin, and prostate cancer, as well as lymphoma, melanoma, and leukemia (Table 2). Vitamin A affects cancer by mediating cell growth arrest, DNA damage, apoptosis, and differentiation [37,38]. A few studies have also indicated that vitamin A deficiency due to poor diet may contribute to the development of certain cancers [39,40,41]. Further, preclinical and clinical studies have shown that retinoids are viable treatments for certain cancers [37,38].

### 2.1. Vitamin A in Acute Promyelocytic Leukemia

Acute promyelocytic leukemia (APL) is due to chromosomal translocation mutation between chromosomes 15 and 17, which results in the promyelocytic leukemia–retinoic acid receptor fusion protein (RARA) [42,43]. This fusion protein inhibits promyelocyte differentiation. Some studies suggest that the treatment of APL cells with all-trans retinoic acid (ATRA) induces promyelocyte differentiation and impairs the function of the fusion protein [44]. However, a few recent studies also indicate that ATRA treatment is not effective, and combinational therapies are required. For example, synergistic effects have been observed with arsenic trioxide and ATRA combination therapy [45]. Similarly, de Almeida et al. [46] showed that the combination of gefitinib with ATRA and ATO increases promyelocyte differentiation specifically in ATRA- and ATO-resistant APL cells. In another study by Li et al. [47], it was shown that ethacrynic acid in combination with ATRA causes differentiation and apoptosis of myeloid leukemia cells through reactive oxygen species-dependent inhibition of MMPs and activation of caspase 3/7. Hu et al. [48] also indicated that a combination of CDK4/6 inhibitor palbociclib with ATRA synergistically sensitizes myeloid leukemia cells to ATRA and decreases the growth of leukemia cells. Xi et al. [49] showed that a combination of salinomycin with ATRA induces apoptosis and differentiation in acute myeloid leukemia cells by enhancing the activation of the WNT/β-Catenin signaling pathway. Thus, these and other studies suggest that combination therapies with ATRA and other drugs are effective in controlling APL [50,51,52].

Some clinical studies also suggest that combination therapies with ATRA and ATO increase the efficacy of APL treatment. For example, a clinical phase-2 survey by Lancet et al. [53] has shown that the combination of gemtuzumab ozogamicin with ATO and ATRA increases its therapeutic efficiency in high-risk APL patients. Similarly, a long-term follow-up phase-2 study by Jen et al. [54] demonstrated that a treatment regimen with ATO-ATRA and gemtuzumab ozogamicin is effective in the treatment of standard- and high-risk APL patients. In another randomized multi-center non-inferiority phase III study by Wang et al. [55], similar efficacy was observed in the ATRA-ATO-treated group and the ATRA-ATO-plus-chemotherapy-treated group among APL patients at all risk levels.

### 2.2. Vitamin A in Non-Melanoma Skin Cancers

Vitamin A and its derivatives are known to regulate proliferation, differentiation, and angiogenesis of skin cells. They are collectively involved in the pathophysiology of various skin conditions, such as pustular psoriasis, acne vulgaris, fine wrinkling, and hyperpigmentation. Further, retinoids are effective in the prevention and treatment of non-melanoma skin cancers (NMSC)—skin cancers not derived from melanocytes [56]. Examples include basal cell carcinoma and cutaneous lymphomas, collectively known as keratinocyte carcinomas (KCs), as well as Kaposi’s sarcoma (KS), among others [39]. A few recent studies have also shown the efficacy of vitamin A derivatives in treating melanomas [57,58].

Retinoids regulate epidermal turnover by inhibiting keratinocyte proliferation. Ramchatesingh et al. [56] showed that inhibition of retinoid receptors or retinoid transport proteins in keratinocytes could stimulate hyperproliferation, whereas overexpression of these proteins inhibits keratinocyte proliferation. Several mechanisms have been proposed, including the stimulation of tumor suppressor genes, the induction of DNA damage and cell cycle arrest, and the inhibition of proliferation-promoting signaling. Some studies also demonstrate that ATRA increases p53 expression and upregulates pro-apoptotic caspases, inducing apoptosis in keratinocytes [59,60,61].

Other studies indicate that physiological responses to retinoids are dose-dependent. For example, high pharmacological doses of retinoids appear to promote epidermal proliferation and thickening, as well as inhibit squamous cell differentiation and keratinization disorders [62,63]. Thus, reports are somewhat contradictory, although it is evident that retinoids exert anti-tumorigenic effects within the skin, regardless of the proliferative effects. One suggested explanation for these phenomena is that the anti-tumorigenic effects (i.e., apoptosis and cell cycle arrest) exert dominance over the pro-tumorigenic effects (i.e., cell proliferation). Importantly, retinoids have been used clinically for decades for skin conditions without increased malignancies, proving their safety [56,64].

Retinoids are not currently FDA-approved for chemoprevention of keratinocyte carcinomas. However, studies have yielded positive results in high-risk patients (i.e., those who are immuno-compromised or those predisposed to KCs). Initially, Kraemer et al. [65] in a clinical trial reported that treating patients with xeroderma pigmentosum with oral isotretinoin (a retinoid commonly used for acne) resulted in a reduction in basal cell carcinoma and cutaneous squamous cell carcinoma formation. After treatment discontinuation, tumors increased by 8.5-fold. This data suggests that the use of off-label retinoids could be beneficial in chemoprevention. Further, epidemiological studies have shown an inverse correlation between retinol intake and skin cancer incidence. A cohort study by Mahamat-Saleh et al. [66] involving 98,995 French women indicated that the use of vitamin A and E supplements is linked to an increased risk of basal cell carcinoma (BCC) and squamous cell carcinoma (SCC). At the same time, beta-carotene supplementation is associated with a higher SCC risk.

Although case studies and preclinical data have shown efficacy in treating KCs with retinoids, large-scale studies have failed to exhibit sufficient sustained responses to treatment. Thus, retinoids are not currently FDA-approved for the treatment of already formed tumors in KC. However, retinoids have proven to be efficacious in treating other malignancies, such as cutaneous T-cell lymphoma [67,68] and Kaposi’s sarcoma (KS) [69,70].

### 2.3. Vitamin A in Melanoma

**Figure 1:**
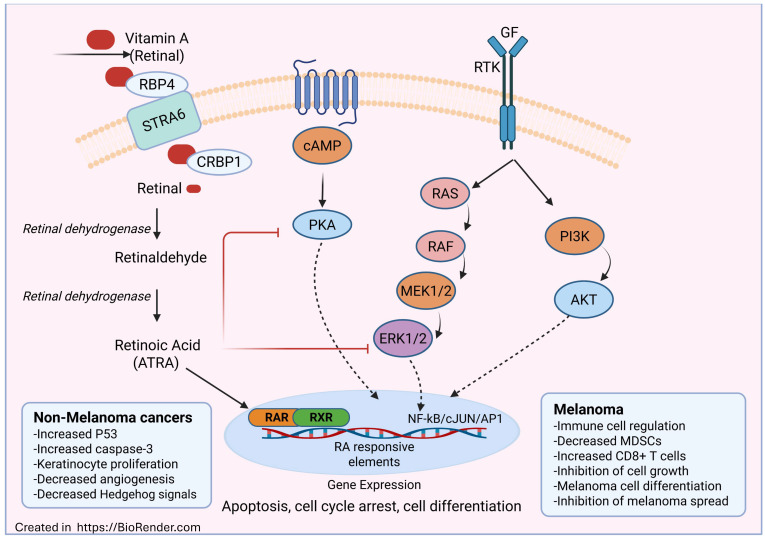
Role of vitamin A in melanoma and non-melanoma cancers. Retinol is transported by RBP4 and internalized through STRA6, which is converted by retinal dehydrogenases into retinaldehyde and subsequently into retinoic acid (ATRA). ATRA activates nuclear receptors such as RAR and RXR. This signaling regulates gene expression involved in apoptosis, cell cycle arrest, and differentiation. In non-melanoma cancers, retinoic acid increases p53 and caspase-3 activity while inhibiting angiogenesis and Hedgehog signaling. In melanoma, it regulates immune responses, suppresses tumor growth, and inhibits metastasis through interactions with the MAPK/ERK and PI3K/AKT pathways. The image was created by using BioRender. (Ramana (2025) https://Biorender.com (accessed on 22 October 2025)).

Preclinical and clinical studies have proven promising in treating melanoma with ATRA (Figure 1). Oliveira et al. [71] indicated that low concentrations of vitamin A could enhance macrophage cytotoxic activity, while higher concentrations reduced it. These results suggest that vitamin A may regulate immune responses and could be developed as a potential preventive strategy for melanoma by increasing the body’s natural defense mechanism. Işlek Köklü et al. [108] examined the combined effects of ATRA and sphingomyelin (SM) in B16-F10 melanoma cells. They found that the combination caused apoptosis and G2/M phase cell cycle arrest, enhanced the expression of pro-apoptotic and tumor-suppressor genes, and suppressed PD-L1 expression and melanoma cell growth. These findings suggest that ATRA and SM together could be a therapeutic strategy for preventing melanoma. Similarly, Jobani et al. [72] showed that allicin sensitizes CD44+ melanoma cells to ATRA-induced cell death. Further, they showed that the combination treatment inhibited melanoma cell proliferation, increased the expression of cyclin D1 and RARβ, and modulated MMP-9 expression. Another study by Kanai et al. [73] showed that the combination of resveratrol with ATRA significantly reduced the expression of stem cell markers, increased differentiation markers, and improved sensitivity to docetaxel. Similarly, ATRA has been shown to enhance the effectiveness of dacarbazine in treating CD117+ melanoma. This combination significantly increased apoptosis and caused cell cycle arrest in the G0/G1 phase compared to dacarbazine alone. This study suggests that ATRA could improve the sensitivity of melanoma cells to dacarbazine. Further, Wang et al. [74] showed that ATRA increases the chemotherapeutic efficacy of paclitaxel by increasing the differentiation of melanoma stem cells. Similarly, ATRA decreases melanoma cells’ resistance to PLX4032 by inhibiting PIN1 [75].

Furthermore, in murine models, the anticancer activity of endogenous and synthetic ATRA has been shown to inhibit cell growth, proliferation and enhance apoptosis. For example, Grace et al. [76] examined the effects of ATRA in preventing metastatic melanoma in the lung and liver in a mouse model. They showed that ATRA treatment prevented tumor growth and restored biochemical markers such as cholesterol and γ-Glutamyl Transferase (GGT) levels. These results suggest that ATRA has anti-metastatic effects against melanoma metastasis. Similarly, another study by Yin et al. [77] demonstrated that topical application of ATRA effectively inhibited B16F10 melanoma growth by enhancing CD8+ T-cell responses but not CD4+ T-cell responses. They showed that the tumor-inhibitory effects of ATRA were partly dependent on CD8+ T cells and were linked to upregulated MHCI expression. Moreover, a recent study by Chen et al. [78] showed that WYC-209 (a novel synthetic retinoid) effectively inhibits the proliferation of tumor-repopulating cells (TRCs) in malignant melanoma and other cancers. In this study, WYC-209 was shown to significantly reduce melanoma lung metastases in mice by activating retinoic acid receptors (RAR) and inducing caspase-3-mediated apoptosis. When used in conjunction with chemotherapeutics, these effects were amplified up to 5 times.

Furthermore, a phase Ib/II clinical trial by Tobin et al. [79] evaluated the safety and efficacy of combining ATRA with pembrolizumab in stage IV melanoma patients. Their results indicate that this combination was well tolerated, with a 71% overall response rate, a 50% complete response rate, and a median progression-free survival of 20.3 months. These findings suggest that targeting myeloid-derived suppressor cells (MDSCs) with ATRA could enhance the efficacy of immunotherapy for melanoma. Similarly, a randomized phase II clinical trial (NCT02403778) evaluated the addition of ATRA to ipilimumab therapy in advanced melanoma patients [80]. The results from this study demonstrate that ATRA reduced the immunosuppressive function of MDSCs and downregulated immunosuppressive genes, suggesting that the combination of ATRA with ipilimumab could be an effective strategy for melanoma treatment. Further, a recent study by Mittal et al. [81] examined the relationship between total vitamin A intake and the risk of cutaneous melanoma (CM) and non-melanoma skin cancer (NMSC) in postmenopausal women. They used data from 52,877 White women over an average follow-up of 17.8 years. The study findings indicate that there is no association between total vitamin A intake and melanoma risk. However, higher dietary vitamin A and beta-cryptoxanthin intake could be linked to a slightly increased risk of NMSC.

### 2.4. Vitamin A in Breast, Lung, and Head and Neck Cancers

Epidemiological studies from the past two decades have demonstrated an inverse relationship between blood vitamin A levels and breast cancer development, suggesting a protective effect against breast cancer development [82]. Anticancer activity of endogenous ATRA was found to correlate with a decrease in the number of mitochondria in breast cancer cells. Further, it inhibited cell proliferation and survival [83]. Peng et al. [84] conducted a nested case–control study within the Nurses’ Health Studies examining the association of both pre-diagnostic plasma metabolites and circulating carotenoids with breast cancer risk. They found that metabolomic signatures for β-carotene and vitamin A were associated with a lower breast cancer risk, indicating that carotenoid supplementation could reduce breast cancer development. Similarly, a recent systematic review and meta-analysis study examined the association between circulating carotenoids and breast cancer risk by analyzing 15 publications with over 20,000 participants [85]. This study found an inverse relationship between higher levels of total carotenoids (α-carotene, β-carotene, β-cryptoxanthin, lycopene, and lutein) and a reduced risk of breast cancer. They specifically indicated that each 10 μg/dL increase in specific carotenoid consumption is associated with a 2–22% lower risk of developing breast cancer [85]. In addition, a few additional studies also suggest that carotenoid supplementation prevents the risk of developing breast cancer [86,87]. Further, a recent study indicated that MCF-7 breast cancer cells responded more effectively to ATRA than MDA-MB-231 cells, showing reduced ER(α), H19, telomerase, PKM2, and LDHA expression, and increased ER(β) and miR-let-7a. On the other hand, MDA-MB-231 cells exhibited gene expression changes without significant alterations in protein or activity. Further, ATRA co-treatment has been shown to reduce glycolytic enzyme expression [88]. Another recent article by Caricasulo et al. [89] demonstrated pre-clinical and clinical studies on ATRA’s direct effects on breast cancer cells, including ATRA-based clinical trials.

**Figure 2:**
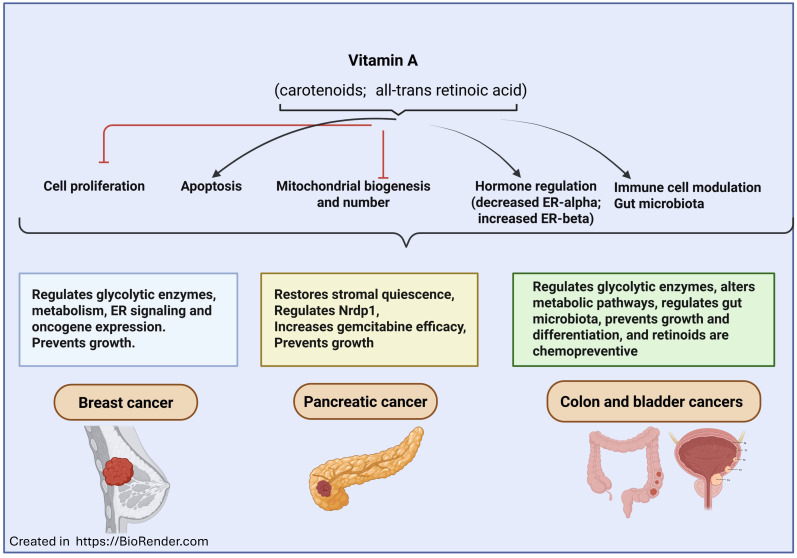
Role of vitamin A in the prevention of breast, pancreatic, colon, and bladder cancers. Vitamin A compounds such as carotenoids and retinoids, specifically all-trans retinoic acid (ATRA), could decrease proliferation, enhance apoptosis, decrease mitochondrial content, modulate hormone and gene expression, and alter the immune cell metabolism specifically in the tumor microenvironment. These processes, along with specific regulation of glycolytic enzymes, signaling pathways, and the microbiome, could mediate vitamin A’s chemopreventive effects in breast, pancreatic, colorectal, and bladder cancers. The image was created by using BioRender. (Ramana (2025) https://Biorender.com (accessed on 26 November 2025)).

Furthermore, recent studies have yielded promising results in treating various other cancers with vitamin A (Figure 2). In vitro and in vivo studies, primarily in mouse models, have shown reduced cell growth and proliferation, and amplified apoptosis, in colorectal, lung, gastric, pancreatic, thyroid, and prostate cancers, as well as neuroblastoma, glioma, and hepatocellular carcinoma, with administration of exogenous ATRA. For example, Xue et al. [97] showed that cigarette smoke-induced vitamin A deficiency could lead to an increase in lung cancer in rats. Similarly, Okayasu et al. [98] showed that vitamin A prevents DSS-induced colon cancer in mice. Further, Luo et al. [99] also examined the role of vitamin A in bladder cancer. They found that vitamin A, by regulating the gut microbiota, helps in the prevention of bladder cancer. A recent narrative review discussed the role of vitamin A and retinoids in bladder cancer, and this study reports that retinoids have significant therapeutic effects in preventing bladder cancer in pre-clinical models [100]. Similarly, chemopreventive effects of vitamin A have been reported by several studies [101,102,103]. Retinoic acid derivative A4-amino-2-(butyrylamino)phenyl (2E,4E,6E,8E)-3,7-dimethyl-9- (2,6,6-trimethyl-1-cyclohexenyl)-2,4,6,8-nonatetraenoate (ABPN) has been shown to prevent pancreatic cancer by regulating Nrdp1 in in vitro and in vivo xenograft mouse models [90]. Further, Chronopoulos et al. [91] showed that ATRA restores the mechanical quiescence of pancreatic stellate cells (PSCs) in pancreatic ductal adenocarcinoma by activating the RAR-β pathway. They also showed that it inhibits cancer cell invasion, suggesting a potential therapeutic strategy for pancreatic adenocarcinoma. Similarly, Kuroda et al. [92] demonstrated that ATRA enhances the effectiveness of gemcitabine in resistant pancreatic cancer cells by upregulating deoxycytidine kinase (dCK). Another study by Wang et al. [93] also indicated that ATRA, by regulating p21-activated kinases, prevents pancreatic cancer. A meta-analytical study by Zhang et al. [94] found that higher dietary vitamin A intake is associated with a reduced risk of pancreatic cancer (RR = 0.839, 95% CI = 0.712–0.988), particularly in case–control and European studies. Another meta-analysis study by Huang et al. [95] also indicated that higher dietary intake of vitamin A, β-carotene, and lycopene is significantly associated with a lower risk of pancreatic cancer. Kocher et al. [96] conducted a phase Ib STARPAC trial using ATRA. In this study, ATRA was safely repurposed as a stromal-targeting agent alongside gemcitabine–nab-paclitaxel in patients with advanced pancreatic ductal adenocarcinoma. The results indicate that the combination showed manageable toxicity, demonstrated stromal modulation, and achieved a median overall survival of 11.7 months for treatment response. Additional studies are required to understand the role of vitamin A in cancers such as thyroid and glioblastoma. A small-scale clinical study by Groener et al. [104] examined the responses induced by retinoic acid-based redifferentiation therapy followed by radioiodine treatment in some patients with radioiodine-refractory papillary thyroid cancer, including those with the BRAF V600E mutation. They found beneficial effects of retinoic acid-mediated redifferentiation therapy. Similarly, Fu et al. [105] showed that ATRA prevents glioblastoma progression by regulating the AKT/mTOR/PPARγ/Plin4 pathway. Another study by Jones et al. [106] showed that ATRA-eluting poly (diol citrate) wafers could prevent glioblastoma. Further, Ye et al. [107] conducted a phase II study in patients with recurrent or metastatic adenoid cystic carcinoma of the head and neck (R/M ACCHN). The subjects were treated with a combination of ATRA and low-dose apatinib after prior anti-angiogenic therapy. The results suggest that ATRA plus apatinib could be a promising therapeutic option for R/M ACCHN. Although some of the above-listed clinical studies showed beneficial effects of ATRA in the treatment of cancers, the sample sizes were small. Large-scale and multicenter studies are required to understand the significance of vitamin A in treating these cancers.

## 3. Vitamin E in Cancer Prevention and Treatment

The vitamin E family is a group of eight lipophilic compounds known as α-tocopherol, β-tocopherol, γ-tocopherol, δ-tocopherol, α-tocotrienol, β-tocotrienol, γ-tocotrienol, and δ-tocotrienol [109]. Tocopherols are primarily synthesized by plants and cannot be produced by humans; therefore, humans mostly obtain Vitamin E from dietary sources. Vitamin E is absorbed from the intestinal mucosa, integrated into chylomicrons, and transported to various peripheral tissues via the lymphatic system. They are then brought into the tissue through lipoprotein-receptor-mediated endocytosis [110]. All forms of vitamin E are potent antioxidants as they prevent the propagation of free radicals in the cell membrane. α-tocopherol is the major form of vitamin E found in humans and has been extensively investigated for its role in cancer prevention [111]. However, large-scale trials have not suggested a link between α-tocopherol and cancer prevention. On the other hand, γ-tocopherol has been suggested to be more potent than α-tocopherol in cancer prevention due to its strong anti-inflammatory properties [112]. For example, an in vitro study found that α-tocopherol was the least effective of vitamin E isomers against a wide range of breast and pancreatic cancer lines. It also suggested that γ- and δ-tocotrienol display a more potent effect when compared to α-tocopherol [113]. Due to inconsistent results with α-tocopherol, recent studies have been more focused on alternate forms of vitamin E in cancer prevention [114,115].

### 3.1. Vitamin E in Colorectal Cancer

There are a few studies that have shown the relationship between dietary intake of tocopherols and their blood levels in the risk of colorectal cancer development [116]. For example, Jang et al. [117] found a strong association between vitamin E supplementation and reduced risk of colon cancer. Further, when compared to a-tocopherol, d-tocopherol and γ-tocopherol have been shown to be significantly effective in preventing the formation of colorectal adenomas in azoxymethane-treated rats [118]. These studies thus suggest that a mixture of tocopherols could potentially have cancer treatment efficacy (Table 3).

Furthermore, a recent study by Falsetti et al. [119] also indicated that natural δ-tocopherol and its derivatives ((δ-Toc)_2_S and (δ-Toc)_2_S_2_) could inhibit cell proliferation and upregulate ERβ expression in ERβ-overexpressing colon cancer HCT8 cells. Similarly, Khalid et al. [120] also showed that natural γ- and δ-forms of tocotrienols (T3s) inhibit CRC cell growth and metastasis. This study indicates that T3s, by regulating telomerase activity as well as immune responses, could act as potential anticancer agents. Additionally, Husain et al. [121] showed that T3s in combination with aspirin inhibited Wnt/β-catenin signaling and reduced the expression of cancer-related proteins in CRC stem cells. Further, they also demonstrated that in an APCmin/+ mouse model, the combination treatment significantly reduced intestinal adenoma formation. This study also suggests the potential of the combination of T3 and aspirin as a preventive strategy for colorectal cancer. Along similar lines, Schlörmann et al. [122] also indicated that long-chain metabolites of vitamin E, such as α-13′-OH and α-13′-COOH, could significantly reduce cell proliferation of premalignant colon cells and demonstrated antioxidant and DNA-protective effects. In contrast, vitamin E showed no growth inhibition. These results suggest that the long-chain metabolites of vitamin E, by regulating ROS scavenging and caspase-independent cell death mechanisms, could act as chemopreventive agents. Another study by Liu et al. [123] also indicated that combining γ-tocopherol with aspirin significantly reduced tumor growth and size in a mouse model of colitis-associated colon cancer. Further, γ-tocopherol also reduced aspirin-induced inflammation and stomach lesions, and the combination showed synergistic effects on CRC growth, probably by modulating the gut microbiota. These findings suggest that γ-tocopherol and aspirin together may offer a more effective and safer chemopreventive strategy for CAC. Similarly, Yang et al. [124] also showed that δ-tocotrienol (δTE) and its metabolite δTE-13′ significantly reduce tumor formation and inflammation in a mouse model of colitis-associated colon cancer, especially by modulating the gut microbiota composition. Bazzaz et al. [125] also showed that a combination of γ-tocopherol with 5-fluorouracil (5-FU) enhanced cytotoxic and pro-apoptotic effects against HT-29 colon cancer cells. They showed that the combination specifically increased apoptosis, induced cell cycle arrest in the sub-G1 phase, and altered expression of cell cycle- and apoptosis-related genes. These results demonstrate that γ-tocopherol supplementation could improve the efficacy of 5-FU in colon cancer chemotherapy. Interestingly, Chen et al. [126] also showed that δ-tocopherol and γ-tocopherol, but not α-tocopherol, significantly reduce colon tumor formation in a humanized mouse model of colon cancer. Further, δ-tocopherol and γ-tocopherol were shown to suppress oxidative/nitrosative stress, indicating their protective role against DNA damage. These findings indicate the superior chemopreventive potential of δ-tocopherol and γ-tocopherol over α-tocopherol in colon cancer therapy.

On the other hand, Zhao et al. [176], in a Mendelian randomization study, investigated if dietary antioxidant vitamins (retinol, carotene, vitamin C, and vitamin E) and oxidative stress biomarkers (GST, CAT, SOD, and GPX) could reduce the risk of colorectal cancer. The genetic data showed no significant association between these antioxidants or biomarkers and CRC risk. These findings suggest that neither increased antioxidant intake nor higher oxidative stress biomarker levels provide protective benefits against colorectal cancer. In a randomized, double-blind, and placebo-controlled study by Raunkilde et al. [177], a combination of d-tecotrienol along with first-line 5-fluorouracil, oxaliplatin, and irinotecan (FOLFOXIRI) treatment for metastatic CRC did not significantly extend the time to first hospitalization or death compared to the placebo. However, fewer patients in the δ-tocotrienol group required oxaliplatin dose reductions, suggesting a potential neuroprotective effect.

### 3.2. Vitamin E in Lung Cancer

The role of vitamin E in lung cancer is not very well explored. However, a few studies indicate its potential chemopreventive efficacy. A recent study by Yoon et al. [127] found that higher plasma levels of total tocopherols were associated with a lower risk of lung cancer, particularly among European Americans, men, current smokers, and patients diagnosed within two years of blood draw. Further, this protective effect has been associated with the form of tocopherol and individual characteristics. A long-term study including 22,781 male smokers found that higher serum a-tocopherol levels were significantly associated with reduced lung cancer risk over 28 years [128]. The protective effect was seen both at baseline and after three years, with stronger associations in younger men and those with shorter smoking histories. Men with initially low vitamin E levels who are on vitamin E supplementation, which increases levels over time, also experienced a lower risk. This data suggests the potential benefit of maintaining adequate vitamin E levels [128].

Similarly, a meta-analysis study by Zhu et al. [129] found that higher dietary vitamin E intake is associated with a reduced risk of lung cancer. Specifically, these results suggest that individuals in the category with the highest intake of vitamin E had a 16% lower risk compared to those in the category with the lowest. Further, they showed that each 2 mg/day increase in vitamin E intake was linked to a 5% reduction in lung cancer risk. These results further support a protective role of dietary vitamin E against lung cancer.

Another study by Wiel et al. [130] showed that long-term antioxidant supplementation with N-acetylcysteine (NAC) and vitamin E promotes KRAS-driven lung cancer metastasis by reducing free heme and stabilizing the transcription factor BACH1. They demonstrated that the inhibition of BACH1 could prevent antioxidant-induced metastasis. Similarly, Rajasinghe et al. [131] also showed that δ-tocotrienol disrupts glutamine metabolism in non-small-cell lung cancer (NSCLC) cells by inhibiting the uptake of glutamine and essential amino acids. Specifically, they have shown that δ-tocotrienol downregulated glutamine transporters ASCT2 and LAT1, and suppressed mTOR pathway proteins, leading to increased apoptosis. The same research group [132] also indicated that δ-tocotrienol inhibits NSCLC metastasis by downregulating the Notch-1/NF-κB/uPA signaling pathway and upregulating miR-451 expression. These results suggest that delta-tocotrienol may have therapeutic potential in preventing NSCLC growth and metastasis by regulating multiple pathways. Similarly, Uchihara et al. [133] showed that α-tocopherol significantly reduces the anti-tumor effectiveness of crizotinib in EML4-ALK-positive NSCLC cells by inhibiting crizotinib-induced apoptosis and blocking its downstream signaling effects. Other vitamin E forms, such as β-, γ-, δ-tocopherol, and α-tocotrienol, did not interfere with crizotinib’s activity. These findings indicate a potential risk associated with combining α-tocopherol supplements with crizotinib therapy in NSCLC patients. However, another study by Daifuke et al. [134] developed vitamin E phosphate (VEP) nucleoside prodrugs to overcome resistance mechanisms in cancer treatment by enabling nucleoside transport-independent delivery of gemcitabine. Two compounds, δ-tocopherol-MP gemcitabine (NUC050) and δ-tocotrienol-MP gemcitabine (NUC052), significantly inhibited tumor growth and extended survival in a mouse model of NSCLC compared to controls. This study suggests that NUC050 and NUC052 are safe and effective alternatives for enhancing gemcitabine-based therapy in NSCLC. Thus, recent studies indicate that higher dietary intake of vitamin E, particularly in the form of γ- and δ-tocopherol or tocotrienols, has been associated with a reduced risk of lung cancer, especially among smokers. On the other hand, high doses of α-tocopherol have shown inconsistent or even harmful effects, including interference with cancer therapies and promotion of metastasis in specific settings. Thus, the role of vitamin E in lung cancer depends on its form and dosage, as well as lifestyle habits.

### 3.3. Vitamin E in Prostate Cancer

A few studies also suggest a role of vitamin E in prostate cancer. Mondul et al. [135] conducted a prospective analysis study on prostate cancer within an a-tocopherol and β-carotene cancer prevention study cohort. In this study, they examined 200 prostate cancer cases (including 100 aggressive) and 200 matched controls using fasting serum collected up to 20 years before diagnosis. This study found that among 626 consistently detected compounds, strong inverse associations were found between several energy and lipid metabolites (particularly inositol-1-phosphate, glycerophospholipids, and fatty acids) and risk of aggressive prostate cancer. Further, higher levels of thyroxine and trimethylamine oxide were associated with increased risk of prostate cancer. Similarly, Antwi et al. [136] conducted a 6-month intervention trial in South Carolina that included 39 men with biochemically recurrent prostate cancer, and assessed plasma carotenoids and tocopherols in relation to PSA levels. They found that higher levels of cis-lutein/zeaxanthin, α-tocopherol, β-cryptoxanthin, and all-trans-lycopene were significantly associated with lower PSA levels. Further, increased α-tocopherol and trans-β-carotene was linked to reduced PSA at 3- and 6-month follow-ups. These results indicate that vitamin E micronutrients may help reduce PSA progression in recurrent prostate cancer.

Wang et al. [137] showed that delta-tocopherol causes cell cycle arrest and apoptosis via an AKT-dependent mechanism in the prostate cancer cell line DU145. Similarly, Yeganehjoo et al. [138] showed the combined effects of δ-tocotrienol and geranylgeraniol on prostate cancer DU145 cells. Both compounds individually suppressed cell growth; however, their combination produced synergistic effects. The combination also induced G1 cell cycle arrest and downregulated HMG CoA reductase and K-RAS protein levels. Another study by Fajardo et al. [139] compared the effects of alpha-tocopherol (AT) and its oxidation product tocopherylquinone (TQ) on prostate cancer cells. They showed that TQ, but not AT, strongly inhibited the growth of androgen-responsive prostate cancer cell lines and suppressed androgen receptor (AR) activity. Further, they found that TQ reduces PSA release, inhibits androgen-responsive gene expression, and downregulates AR protein. These results suggest that TQ represents a potent anti-androgenic agent in androgen-responsive prostate cancer cells. Another study by Wang et al. [141] showed that δ-tocopherol (δ-T) could prevent prostate cancer in Ptenp−/− mice by decreasing AKT activity and increasing apoptosis in prostate lesions. Further, this study also showed that δ-T, but not α-T, suppresses prostate tumor development primarily by inhibiting AKT signaling. Similarly, another study by the same group also showed that d-tocopherol is the most potent form of vitamin E in preventing prostate cancer [141]. Interestingly, Sato et al. [142] also examined the combined effects of δ-tocotrienol (δ-T3) and γ-tocopherol on androgen-dependent prostate cancer LNCaP cells. The results indicate that δ-T3 alone induced G1 cell cycle arrest, while the combination with γ-tocopherol enhanced anticancer activity by also causing G2/M arrest and significantly inhibited prostate cancer cell growth. These results suggest that combining δ-T3 with γ-tocopherol provides a stronger therapeutic effect than either compound alone. Similarly, Fontana et al. [143] showed that δ-tocotrienol (δ-TT) exerts strong cytotoxic and pro-apoptotic effects in castration-resistant prostate cancer (CRPC) cells. They tested two cell lines. In PC3 cells, they observed that δ-TT activated ER stress and autophagy, leading to antitumor actions. In DU145 cells, δ-TT activated only ER stress. Further, in both these cell types, δ-TT induced vacuolation, JNK/p38 signaling, and paraptosis induction. In another study, Moore et al. [144] compared the effects of γ-tocopherol and AT on prostate cancer cell growth. The results indicate that γ-tocopherol, but not AT, activated the RAF/RAS/ERK pathway and upregulated phospho-c-JUN. Further, γ-tocopherol also triggered caspase-9 and -3 activation and induced apoptosis in both androgen-sensitive and androgen-independent cells. Tang et al. [145] also showed that γ-tocopherol causes apoptosis in prostate cancer cells by targeting the angiopoietin-1 and Tie-2 signaling pathways. Further, tocotrienol treatment in a prostate cancer bone metastasis model (VCaP-luc xenografts in nude mice) indicated that tocotrienol significantly inhibited tumor growth. Further, this growth inhibition was linked to increased CDK inhibitors p21 and p27, increased H3K9 acetylation, and decreased histone deacetylase expression [146]. A recent study by Sun et al. [147] examined whether different natural forms of vitamin E vary in their ability to enhance the effectiveness of chemotherapy. By using paclitaxel as a model drug and breast/prostate cancer cells, they found that the chemosensitization effect of vitamin E is form-dependent. δ-tocotrienol (δ-T3) was found to be most effective at sensitizing cancer cells, most likely by suppressing PDL1-mediated tumor-promoting signaling. These findings suggest that δ-T3 is the most potent among vitamin E forms for improving taxane-based cancer therapy. Although most studies on vitamin E and prostate cancer have been conducted in pre-clinical settings, no significant clinical studies have been conducted in the last ten years. A few clinical studies conducted in the past suggest its potential but warrant further investigations. For example, a study published in 2000 compared 117 male subjects who developed prostate cancer against 233 matched control subjects. All of them provided toenail and plasma samples for assays of selenium, α-, and γ- tocopherol [148]. The results indicate a declining risk of prostate cancer with increasing levels of α-tocopherol, and a fivefold reduction in prostate cancer risk with high levels of γ-tocopherol. These findings could be an indicator of the anti-prostate-cancer properties of vitamin E. However, the Selenium and Vitamin E Cancer Prevention Trial (SELECT), published in 2009, suggests that vitamin E supplementation is a risk factor for prostate cancer, contradicting the results of the 2000 study [149]. In 2009, SELECT randomized 35,533 men, for whom there was no suspicion of prostate cancer, into four groups with daily dietary supplementation of selenium, vitamin E, both, or a placebo, and followed up with the subjects from 2001 to 2004 [149]. This trial found that there was no correlation between vitamin E and prostate cancer prevention. Nevertheless, the updated SELECT study in 2011 found that there was a significant increase in risk between dietary supplementation of vitamin E and developing prostate cancer among healthy men [150].

### 3.4. Vitamin E in Breast Cancer

Like in prostate cancer, vitamin E’s role in breast cancer also depends on its form. For example, tocotrienols, particularly δ- and γ-tocotrienol, have been shown to have the most promising anticancer and chemosensitizing effects, while α-tocopherol alone has limited benefits. Further, most of the studies published are preclinical, with only a few clinical reports. Drotleft et al. [151] showed that oxidized tocotrienols prevent cell growth in MCF-7 breast cancer cells. Another study by Alawin et al. [152] reported that γ-tocotrienol inhibits the growth of HER2-positive breast cancer cells by disrupting HER2 signaling within the lipid raft microdomains. They also showed that γ-tocotrienol accumulates explicitly in microdomains, leading to reduced HER2 dimerization and decreased cancer cell viability. Similarly, Ahmed et al. [153] showed that γ-tocotrienol inhibits the growth of breast cancer cells (MDA-MB-231 and T-47D) without affecting normal mammary epithelial cells (MCF-10A). They showed that g-toc suppressed canonical Wnt/β-catenin signaling, reversed epithelial–mesenchymal transition (EMT), and reduced cancer cell motility. Interestingly, Diao et al. [154] showed that vitamin E supplementation accelerated breast cancer growth in both mouse models and MCF7 cell cultures. In this study, treatment with vitamin E or Trolox reduced intracellular ROS levels and suppressed p53 expression, leading to increased cancer cell proliferation. This study thus suggests that vitamin E promotes breast cancer progression by lowering ROS and inhibiting p53. Further, γ-tocotrienol has been shown to reverse multidrug resistance in breast cancer MCF-7/Adr cells [155]. γ-tocotrienol has been shown to suppress P-glycoprotein (P-gp) expression, leading to greater doxorubicin accumulation, G2/M arrest, and apoptosis [156]. Similarly, different tocopherol forms such as γ- and δ-tocopherols, as well as a γ-tocopherol-rich mixture (γ-TmT), have been shown to significantly reduce tumor volume and estrogen-related gene expression in MCF-7 cells in vitro and in mouse models [157]. Ding et al. [155] showed that γ-tocotrienol can reverse multidrug resistance in breast cancer MCF-7/Adr cells by targeting the NF-κB/P-gp axis. Another study by Ye et al. [158] showed that a vitamin E-rich nanoemulsion promoted Th1 cytokine secretion, reduced Th2 cytokines, and induced higher apoptosis in breast cancer cells. Further, in an in vivo model, the study showed significant tumor suppression by both Taxol and low-vitamin E nanoemulsions at a reduced paclitaxel dose. This study suggests that by reducing the immune response, vitamin E could enhance paclitaxel efficacy. Several other studies have also shown that vitamin E could improve the effectiveness of chemotherapeutic drugs [159,160,161].

Furthermore, a randomized pilot study by Schmidt et al. [162] examined a vitamin E-loaded nanoparticle cream in 40 women with breast cancer undergoing radiotherapy. This study indicates that while all patients developed radiodermatitis, the vitamin E cream delayed its onset and reduced mild inframammary erythema in women who did not receive a radiation boost. These results suggest a potential protective benefit of vitamin E cream. Similarly, another randomized controlled trial by Long et al. [163] compared Sanyrene^®^ with DaBao^®^ (hyaluronic acid + vitamin E) to prevent radiation dermatitis in breast and head and neck cancer patients. Sanyrene significantly reduced the incidence of ≥grade 2 dermatitis (22% vs. 67.3%) and improved skin-related quality-of-life scores. Another randomized controlled study by Moustafa et al. [164] evaluated the cardioprotective effect of vitamin E and levocarnitine in breast cancer patients receiving doxorubicin–cyclophosphamide chemotherapy. Patients given these drugs showed significantly lower levels of B-type natriuretic peptide and creatine kinase. This study suggests that vitamin E, along with levocarnitine, could be an effective prophylactic agent against doxorubicin-induced cardiotoxicity. Further, a phase II trial by Kjaer et al. [165] investigated delta-tocotrienol with standard neoadjuvant therapy in 80 women with newly diagnosed breast cancer. This study indicates that the addition of delta-tocotrienol did not improve response rates or reduce adverse events compared to standard treatment alone. This study suggests that delta-tocotrienol adds no clinical benefit in this setting.

### 3.5. Vitamin E in Pancreatic Cancer

Vitamin E’s role in pancreatic cancer is not well studied. There are very few studies that indicate that it has beneficial effects in preventing pancreatic cancer. Husain et al. [166] suggested that δ-tocotrienol selectively targets pancreatic ductal adenocarcinoma (PDAC) stem-like cells. δ-tocotrienol was shown to reduce stem cell viability and expression of stemness markers (Oct4, Sox2). It also inhibited migration, invasion, and angiogenesis pathways. Further, in a mouse xenograft model, δ-tocotrienol significantly suppressed growth and metastases, including in gemcitabine-resistant PDAC stem-like cells. These results indicate δ-tocotrienol’s potential as a safe and effective agent for preventing PDAC progression and metastasis. Another study by Palau et al. [167] showed that γ-tocotrienol induces apoptosis in pancreatic cancer cells by altering ceramide metabolism and transport. They demonstrated that γ-tocotrienol stimulated ceramide synthesis in the ER and plasma membrane while inhibiting its conversion to sphingomyelin and glucosylceramide through suppression of CERT. These findings suggest that γ-tocotrienol’s anticancer effects are partly mediated through ceramide upregulation and regulation of ceramide transport pathways.

Francois et al. [168] demonstrated that δ-tocotrienol enhances TRAIL-induced apoptosis in pancreatic cancer cells by promoting degradation of the anti-apoptotic protein c-FLIP. δ-tocotrienol was shown to increase c-FLIP ubiquitination and sensitize cells to caspase-8-dependent apoptosis. This study also indicates that only the bioactive forms of vitamin E (δ-, γ-, and β-tocotrienol) showed this effect, but not α-tocotrienol. Further, Tang et al. [169] developed α-tocopherol–conjugated polycation nanoparticles (PAMD-TOC) to enhance siRNA delivery targeting STAT3 in pancreatic ductal adenocarcinoma (PDAC). When compared to parent PAMD nanoparticles, PAMD-TOC/siSTAT3 showed better uptake, tumor penetration, and accumulation. This led to inhibition of cancer growth, migration, and metastasis in both mouse and human PDAC models. Similarly, Behera et al. [170] developed human serum albumin-based nanoparticles (HSA NPs) loaded with α-tocopherol succinate (TOS) and gemcitabine (GEM) to improve pancreatic cancer therapy. The combination of nanoparticles showed synergistic cytotoxicity in MIA PaCa-2 cells. Furthermore, these results suggest that TOS-HSA and GEM-HSA nanoparticles are a promising micronutrient-based combination strategy for pancreatic cancer treatment. In addition, Pereira-Silva et al. [171] developed vitamin E succinate–gemcitabine (VES-GEM) prodrug micelles using Pluronic^®^ F68 and F127 carriers to overcome the poor stability and delivery of gemcitabine in pancreatic cancer. The VES-GEM conjugate increased hydrophobicity, enabling >95% encapsulation efficiency and stable micelle formation. Pluronic^®^ F127/VES-GEM micelles showed superior drug release and potent pancreatic cancer prevention. In another study by the same group, Pereira-Silva et al. [172] developed a nanocarrier system for controlling pancreatic cancer. They linked gemcitabine (GEM) with vitamin E succinate (VES) and encapsulated it within d-α-tocopheryl polyethylene glycol succinate (TPGS) micelles. The resulting optimized micelles were shown to prevent the growth of BxPC3 pancreatic cancer cells, indicating a promising biomimetic nanosystem for advanced PC therapy.

A few significant clinical studies have been reported in the last 10 years on pancreatic cancer and vitamin E. A phase I “window-of-opportunity” trial by Springett et al. [173] examined δ-tocotrienol in 25 patients with pancreatic ductal neoplasia before surgery. This study suggests that daily oral doses up to 3200 mg were well tolerated, and significant induction of tumor cell apoptosis was observed at doses between 400 and 1600 mg/day. These results suggest that δ-tocotrienol is a safe, biologically active compound for prevention of pancreatic cancer. Similarly, Mahipal et al. [174] also showed that δ-tocotrienol is chemo-preventive.

Furthermore, Li et al. [175] conducted an extensive case–control study to investigate how overall meat intake was not linked to pancreatic ductal adenocarcinoma (PDAC) risk. Importantly, this study suggests that higher dietary intake of vitamin C or E was inversely associated with PDAC risk and increased risk from 2-amino-3,4,8-trimethylimidazo[4,5-f]quinoxaline exposure. These findings suggest that vitamins C and E may provide protective effects against mutagen-related pancreatic cancer.

**Figure 3:**
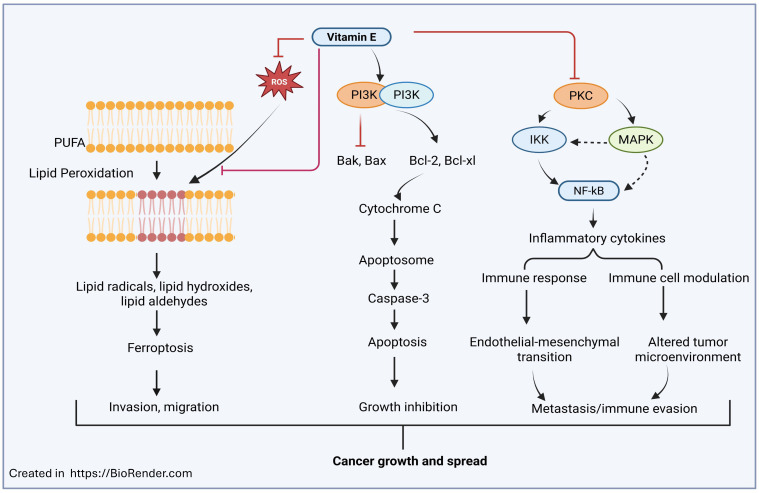
Role of vitamin E in cancer prevention and treatment. Vitamin E could inhibit reactive oxygen species (ROS) and lipid peroxidation in polyunsaturated fatty acids (PUFAs) and thus prevent the formation of lipid radicals and ferroptosis, which cause tumor invasion and migration. Further, through PI3K and PKC signaling pathways, vitamin E enhances pro-apoptotic proteins (Bak, Bax) and suppresses anti-apoptotic factors (Bcl-2, Bcl-xl), leading to cytochrome C release and caspase-3-mediated apoptosis. Additionally, vitamin E can also downregulate NF-κB-dependent inflammatory cytokine production and immune cell modulation, which eventually leads to cancer growth and spread. The image was created by using BioRender. (Ramana (2025) https://Biorender.com (accessed on 22 October 2025)).

The studies mentioned above have demonstrated mixed results regarding α-tocopherol and cancer prevention (Figure 3). Some studies have shown the potential of other forms of vitamin E, such as mixed α- and γ-tocopherols for colorectal cancer; α-, β-, γ-, and δ-tocopherols for lung cancer; and γ- and δ-tocotrienol for pancreatic cancer. These alternate forms of tocopherols and tocotrienols may possess high antioxidant properties. They could be linked to the prevention and treatment of cancers, either in combination with or separately from α-tocopherol. Some studies also suggest that a mixture of tocopherol and/or tocotrienols is more effective than a single tocopherol at suppressing inflammation, and it may play a role in cancer prevention. Large-scale trials for other forms of tocopherols and tocotrienols could be beneficial in investigating a possible link between vitamin E supplementation and cancer prevention or treatment. More studies are needed to understand the role of non-α-tocopherol forms of the vitamin E family in cancer prevention and treatment.

## 4. Conclusions and Future Perspectives

Although vitamins A and E have long been studied for their roles in cancer progression and prevention, their effects depend on the form of the vitamins and the type of cancer. Vitamin A primarily acts through its active retinoid metabolites. They can bind nuclear receptors and could alter epithelial differentiation, immune cell regulation, and cell cycle control. Further, several studies have also shown that retinoid signaling could help to preserve epithelial integrity, reduce inflammation, and promote apoptosis in transformed cells [178,179,180]. Moreover, the potential clinical use of pharmacologic retinoids, which have clear therapeutic value in specific cancers such as acute promyelocytic leukemia, has been well reported [181,182]. However, the preventive role of vitamin A in solid tumors such as lung, colon, and breast is not consistent. The expected outcomes are influenced by dose, receptor expression, and genetic variations. Like many vitamins, both deficiency and excess of vitamin A could cause some risks. For example, a deficiency of vitamin A could impair epithelial barriers and immune defense [183]. At the same time, high-dose supplementation of vitamin A can cause toxicity, teratogenic effects, and tumor-promoting effects due to receptor desensitization [184,185].

Similarly, Vitamin E represents a family of isoforms of tocopherols and tocotrienols, which differ in their bioavailability and biological actions. Further, some preclinical studies have demonstrated that vitamin E isoforms could regulate lipid signaling and tumor cell metabolism. Specifically, tocotrienols have been shown to exhibit pleiotropic anticancer effects through regulating cholesterol biosynthesis and oncogene signaling [186]. Similarly, studies with α-tocopherol have shown no or even harmful effects when compared to γ- and δ-tocopherols. These results suggest that isoform selection, dose, and overall nutrient status could determine isoforms’ chemopreventive nature [187]. Further, over-supplementation of a single isoform could negate the other isoforms and reduce their preventive effects. Most of these recent studies suggest that mixed vitamin E isoforms, rather than a single isoform, could be more protective within the dose limitations.

Thus, recent studies strongly suggest that the cancer progression or prevention status of vitamins A and E is most likely dependent on several factors. These factors could be genetic background, dose response, nutritional status and metabolism, and biological actions, including effects on the gut microbiome. Further, appropriate intake of vitamins A and E through a balanced diet is beneficial. At the same time, deficiency or excess may be harmful and could sometimes alter biological reactions and signaling pathways, leading to tumor progression. Identifying specific isoforms of these vitamins and understanding their mechanism of action could facilitate additional large-scale clinical studies. Similarly, identifying and developing delivery systems that can target particular cancers with the correct dose could be a safer and more effective preventive measure than indiscriminate supplementation of these vitamins.

Further studies are also required to understand the pharmacology of retinoid subtypes and vitamin E isoforms. Understanding how supplementation with these vitamin isoforms expresses specific cancer biomarkers and regulates metabolic pathways leading to chemoprevention is crucial for future chemopreventive strategies. Vitamin E plays a significant role in the prevention of lipid peroxidation [188], and its link to cancer prevention or therapy requires further studies.

Recently, several combinational studies have been conducted using vitamin A or E, along with known chemopreventive drugs or other agents [42,43,44,45,46,47,48,49,50,51,52,53,54,55]. Some studies also combined multiple isoforms of vitamins E and examined their potential chemopreventive role [166,167,168,169,170,171,172,173,174,175]. Additional combinational studies, such as those on retinoids plus epigenetic modulators or tocotrienols plus EGFR inhibitors, could help to develop strong chemopreventive responses. These combinations, paired with nanocarrier delivery systems, could further improve tumor selectivity and especially minimize side effects. Additional studies are needed to integrate next-generation sequencing technologies to identify specific biomarkers. Studies on nutrigenomics, proteomics, and microbiomics could also further advance our understanding of how these vitamins could be more beneficial. Similarly, well-integrated studies on identifying polymorphisms in carotenoid cleavage enzymes, retinoid turnover pathways, or vitamin E transport proteins could also help us understand tumor progression activities.

Furthermore, a better diet, a healthy lifestyle, and adequate but not excessive vitamin supplementation could support improved human health. Further, to avoid unwanted pathological consequences, vitamin supplementation should be individualized based on the body’s oxidative stress responses, and most importantly, physician recommendations are necessary [189,190]. Ultimately, the promise of vitamins A and E in cancer prevention does not depend on universal supplementation but on precision approaches that consider isoform types, metabolic background, and routine diet. Thus, vitamins are not simple antioxidant supplements, but as members of bioactive signaling molecules, they can significantly influence human health and disease. Additional studies on these vitamins’ mechanistic potential to control cancer progression will have substantial therapeutic benefits.

## Figures and Tables

**Table 1:** Vitamin A and E dietary sources, as well as effects of deficiency and overdose.

Vitamin	Dietary Sources	Key Biochemical Roles	Deficiency	Overdose/Toxicity
**Vitamin A** (Retinoids; Carotenoids)	Liver, fish oils, egg yolk, butter, fortified milk Provitamin A carotenoids: carrots, spinach, sweet potatoes	Acts as a cofactor in synthesis of rhodopsin for low-light vision Regulates gene transcription via retinoic acid receptor signaling Supports epithelial differentiation and immune function	Night blindness (nyctalopia) due to defective rhodopsin regeneration Xerophthalmia, and keratomalacia due to epithelial metaplasia Impaired immunity, growth retardation, and infertility	Acute toxicity: nausea, vomiting, vertigo, and increased intracranial pressure Chronic toxicity: hepatomegaly, alopecia, skin desquamation, and teratogenicity
**Vitamin E** (Tocopherols; Tocotrienols)	Vegetable oils, nuts, seeds, green leafy vegetables	Acts as an antioxidant, prevents oxidative damage Protects polyunsaturated fatty acids from peroxidation Maintains integrity of RBC membranes and neural tissues	Hemolytic anemia (in premature infants) due to membrane instability Neurological defects: may cause ataxia, peripheral neuropathy, and loss of proprioception	Generally rare, but very high doses could interfere with vitamin K-dependent clotting, causing increased bleeding risk May also cause fatigue and gastrointestinal upset

**Table 2:** Significance of vitamin A in cancer prevention and treatment; key mechanisms and findings.

Cancer Type	Mechanisms	Key Results	References
Acute promyelocytic leukemia	ATRA overcomes PML-RARα differentiation block, enhances apoptosis, decreases MMP and increases caspase-3/7, sensitizes CDK4/6 inhibition, and modulates WNT/β-catenin with salinomycin	ATRA induces promyelocyte differentiation; combining it with ATO, GO, gefitinib, ethacrynic acid, palbociclib, and salinomycin could improve its efficacy	[42,43,44,45,46,47,48,49,50,51,52,53,54,55]
Non-melanoma skin cancers (BCC/SCC), CTCL; Kaposi’s sarcoma	Increases retinoid receptor/transport signaling, decreases keratinocyte proliferation, increases p53 and pro-apoptotic caspases, causes cell cycle arrest, and inhibits angiogenesis	Chemoprevention in high-risk patients; mixed epidemiological data (may be associated with BCC/SCC risk); clinical use in CTCL/KS	[56,57,58,59,60,61,62,63,64,65,66,67,68,69,70]
Melanoma	ATRA can cause apoptosis and G2/M cell cycle arrest; decrease PD-L1, PIN1, and stemness markers; increase differentiation, CD8^+^ T-cell responses, and activate RAR and caspase-3	ATRA synergizes with SM, allicin, and resveratrol, and enhances docetaxel, dacarbazine, and paclitaxel; WYC-209 reduces metastasis; ATRA plus pembrolizumab increases ORR; and ATRA plus ipilimumab reduces MDSC function	[71,72,73,74,75,76,77,78,79,80,81]
Breast cancer	Endogenous ATRA is anti-proliferative, decreases metabolic reprogramming, regulates ER signaling, and increases RARβ	Higher carotenoids/vitamin A are associated with lower risk of BC; ATRA reduces proliferation and survival	[82,83,84,85,86,87,88,89]
Pancreatic ductal adenocarcinoma	RAR-β activation restores PSC quiescence, decreases cancer cell invasion, increases chemo-sensitivity to gemcitabine, and regulates the PAK pathway	ATRA plus gemcitabine–nab-paclitaxel is safe with stromal modulation; meta-analysis studies link higher dietary vitamin A/β-carotene to lower risk of pancreatic cancer	[90,91,92,93,94,95,96]
Other cancers (colon, lung, thyroid and glioblastoma)	Increases cell differentiation and apoptosis, and decreases cell proliferation, immune cell modulation, and the AKT/mTOR/PPARγ/Plin4 axis in glioblastoma	Prevents DSS-colon cancer in mice; deficiency increases smoke-induced lung cancer and promotes gut microbiota-mediated bladder cancer protection; RA redifferentiation benefits the thyroid; ATRA-eluting wafers prevent glioblastoma	[97,98,99,100,101,102,103,104,105,106,107]

**Table 3:** Significance of vitamin E in cancer prevention and treatment; key mechanisms and findings.

Cancer Type	Mechanism of Action	Key Results	References
**Colorectal Cancer** γ-, δ-tocopherol; γ-, δ-tocotrienol; vitamin E metabolites (α-13′-OH, α-13′-COOH)	Inhibits cell proliferation and adenoma formation, upregulates ER-β expression, inhibits Wnt/β-catenin signaling along with aspirin, regulates telomerase activity and immune responses, induces reactive oxygen species (ROS) scavenging and caspase-independent cell death, and suppresses oxidative and nitrosative stress.	δ- and γ-tocopherols prevent colon tumors, γ-tocopherol plus aspirin reduces inflammation and tumor burden, δ-tocotrienol modulates the gut microbiota and reduces colitis-associated cancer, vitamin E metabolites protect DNA from ROS, and its combination with 5-FU enhances apoptosis.	[117,118,119,120,121,122,123,124,125,126]
**Lung Cancer** α-, β-, γ-, δ-tocopherol; δ-tocotrienol	Protects against ROS damage and downregulates KRAS-driven metastasis; δ-tocotrienol inhibits glutamine metabolism and mTOR pathway, increases miR-451, and reduces metastasis; and vitamin E phosphate prodrugs such as NUC050/NUC052 enhance gemcitabine efficacy.	Higher plasma tocopherol levels reduce lung cancer risk in smokers and men, δ-tocotrienol induces apoptosis and reduces NSCLC growth, α-tocopherol could interfere with crizotinib efficacy, and NUC050/052 prodrugs prolong survival of NSCLC mice.	[127,128,129,130,131,132,133,134]
**Prostate Cancer** α-, γ-, δ-tocopherol; δ-, γ-tocotrienol	Induces apoptosis and cell cycle arrest (G1/G2-M) via AKT inhibition, downregulates HMG-CoA reductase and K-RAS, suppresses androgen receptor (AR) signaling, activates ER stress and JNK/p38, inhibits angiopoietin-1/Tie-2 and HDAC expression, and enhances chemotherapy sensitivity through PD-L1 suppression.	δ-Tocopherol and δ-tocotrienol are most potent in reducing AKT activity and inducing apoptosis, γ-tocopherol promotes apoptosis via caspase-9 and -3, and δ-T3 + γ-tocopherol synergistically inhibit LNCaP growth. Some clinical trials (SELECT 2009, 2011) show no prevention benefit.	[135,136,137,138,139,140,141,142,143,144,145,146,147,148,149,150]
**Breast Cancer** γ-, δ-tocotrienol; γ-, δ-tocopherol; α-TOS	Inhibits HER2 signaling and lipid raft formation, suppresses Wnt/β-catenin and reverses EMT, induces apoptosis and cell cycle arrest, overcomes multidrug resistance, increases Th1 and decreases Th2 cytokines, improves paclitaxel efficacy via nano-formulations, and protects against radiodermatitis and doxorubicin-induced cardiotoxicity.	γ- and δ-tocotrienols inhibit breast cancer growth and metastasis, vitamin E nano-emulsions and creams improve chemo/radiotherapy tolerance, and some oxidized tocotrienols show anti-proliferative activity.	[151,152,153,154,155,156,157,158,159,160,161,162,163,164,165]
**Pancreatic Cancer** γ-, δ-tocotrienol; α-, δ-tocopherol succinate; α-tocopherol conjugates	Targets cancer stem-like cells; inhibits migration, invasion, and angiogenesis; modulates ceramide metabolism; promotes TRAIL-induced apoptosis via c-FLIP degradation; enhances siRNA or gemcitabine delivery through tocopherol-based nanocarriers; and induces tumor apoptosis in pre-surgical patients.	δ-Tocotrienol suppresses PDAC stemness and metastasis, γ-tocotrienol promotes apoptosis via ceramide signaling, α-tocopherol succinate nanocarriers potentiate gemcitabine, δ-tocotrienol was safe and pro-apoptotic in a phase-I trial, and high vitamin E intake inversely correlates with PDAC risk.	[166,167,168,169,170,171,172,173,174,175]
**Others** α-, γ-, δ-tocopherol; α-, γ-, δ-tocotrienols	Antioxidant and anti-inflammatory effects reduce ROS and NF-κB activation, and also modulate immune responses and cell differentiation, and these effects are form-dependent (non-α forms > α-tocopherol).	γ- and δ-isomers exhibit stronger anticancer and anti-inflammatory properties, and α-tocopherol is sometimes neutral or adverse; combination of tocopherols and tocotrienols shows additive chemopreventive benefit.	

## Data Availability

No new data were created or analyzed in this study. Data sharing is not applicable to this article.

## References

[1] Andrès E., Lorenzo-Villalba N., Terrade J.E., Méndez-Bailon M. (2024). Fat-Soluble Vitamins A, D, E, and K: Review of the Literature and Points of Interest for the Clinician. J. Clin. Med..

[2] Siener R., Machaka I., Alteheld B., Bitterlich N., Metzner C. (2020). Effect of Fat-Soluble Vitamins A, D, E and K on Vitamin Status and Metabolic Profile in Patients with Fat Malabsorption with and without Urolithiasis. Nutrients.

[3] English K., Uwibambe C., Daniels P., Dzukey E. (2025). Scoping Review of Micronutrient Imbalances, Clinical Manifestations, and Interventions. World J. Methodol..

[4] Savarino G., Corsello A., Corsello G. (2021). Macronutrient Balance and Micronutrient Amounts through Growth and Development. Ital. J. Pediatr..

[5] Omer E., Chiodi C. (2024). Fat Digestion and Absorption: Normal Physiology and Pathophysiology of Malabsorption, Including Diagnostic Testing. Nutr. Clin. Pract..

[6] Munteanu C., Mârza S.M., Papuc I. (2024). The Immunomodulatory Effects of Vitamins in Cancer. Front. Immunol..

[7] Panda P.K., Saraf S., Verma A., Jain A., Bidla P.D., Raikwar S., Kumari P., Jain S.K. (2024). Role of Vitamins in Therapeutic and Targeting Approaches for Prostate Cancer: An Overview. Curr. Drug Targets.

[8] Vo H.V.T., Nguyen Y.T., Kim N., Lee H.J. (2023). Vitamin A, D, E, and K as Matrix Metalloproteinase-2/9 Regulators That Affect Expression and Enzymatic Activity. Int. J. Mol. Sci..

[9] Królikowska K., Kiślak J., Orywal K., Zajkowska M. (2025). Vitamins in the Pathogenesis of Prostate Cancer: Implications for Prevention and Therapeutic Support. Int. J. Mol. Sci..

[10] Sasaki Y., Tokumura K., Yoshimoto M., Hinoi E. (2024). Association between Fat-Soluble Vitamin Metabolic Process and Glioma Progression. Biol. Pharm. Bull..

[11] Lin X., Wang Y., Zhang T., Pu X. (2024). Fat-Soluble Vitamins and Lung Cancer: Where We Are?. Recent Pat. Anticancer Drug Discov..

[12] Mekky R.Y., Elemam N.M., Eltahtawy O., Zeinelabdeen Y., Youness R.A. (2022). Evaluating Risk: Benefit Ratio of Fat-Soluble Vitamin Supplementation to SARS-CoV-2-Infected Autoimmune and Cancer Patients: Do Vitamin-Drug Interactions Exist?. Life.

[13] Palanca A., Ampudia-Blasco F.J., Real J.T. (2022). The Controversial Role of Vitamin D in Thyroid Cancer Prevention. Nutrients.

[14] Kim J.A., Jang J.H., Lee S.Y. (2021). An Updated Comprehensive Review on Vitamin A and Carotenoids in Breast Cancer: Mechanisms, Genetics, Assessment, Current Evidence, and Future Clinical Implications. Nutrients.

[15] De Flora S., Bagnasco M., Vainio H. (1999). Modulation of Genotoxic and Related Effects by Carotenoids and Vitamin A in Experimental Models: Mechanistic Issues. Mutagenesis.

[16] Talib W.H., Ahmed Jum’AH D.A., Attallah Z.S., Jallad M.S., Al Kury L.T., Hadi R.W., Mahmod A.I. (2024). Role of vitamins A, C, D, E in cancer prevention and therapy: Therapeutic potentials and mechanisms of action. Front Nutr..

[17] Misotti A.M., Gnagnarella P. (2013). Vitamin Supplement Consumption and Breast Cancer Risk: A Review. Ecancermedicalscience.

[18] Darouei B., Bohn T., Vahid F., Amani-Beni R., Haghjooy Javanmard S., Zendehdel K., Abdollahpour I. (2025). Dietary Carotenoids and Breast Cancer Risk: Evidence from a Large Population-Based Incident Case-Control Study. Nutr. Metab..

[19] Wen Y., Yang X., Huang Y. (2025). Associations between vitamins intake and risk of cancer in United States adults: 2003 to 2016 National Health and Nutrition Examination Survey. Front. Nutr..

[20] Frost Z., Bakhit S., Amaefuna C.N., Powers R.V., Ramana K.V. (2025). Recent Advances on the Role of B Vitamins in Cancer Prevention and Progression. Int. J. Mol. Sci..

[21] Rao S., Li T., Li X., Hou L., Sun W. (2025). Vitamin A and Its Related Diseases. Food Sci. Nutr..

[22] Vašková J., Stupák M., Vidová Ugurbaş M., Židzik J., Mičková H. (2025). Therapeutic Uses of Retinol and Retinoid-Related Antioxidants. Molecules.

[23] Dawson M.I. (2000). The Importance of Vitamin A in Nutrition. Curr. Pharm. Des..

[24] Harrison E.H. (2022). Carotenoids, β-Apocarotenoids, and Retinoids: The Long and the Short of It. Nutrients.

[25] Steinhoff J.S., Lass A., Schupp M. (2021). Biological Functions of RBP4 and Its Relevance for Human Diseases. Front. Physiol..

[26] Swigris J., Widjaja-Adhi M.A.K., Golczak M. (2025). Retinoid Dynamics in Vision: From Visual Cycle Biology to Retina Disease Treatments. Pharmacol. Ther..

[27] Hong J.D., Salom D., Kochman M.A., Kubas A., Kiser P.D., Palczewski K. (2022). Chromophore Hydrolysis and Release from Photoactivated Rhodopsin in Native Membranes. Proc. Natl. Acad. Sci. USA.

[28] Abadie R.B., Staples A.A., Lauck L.V., Dautel A.D., Spillers N.J., Klapper R.J., Hirsch J.D., Varrassi G., Ahmadzadeh S., Shekoohi S. (2023). Vitamin A-Mediated Birth Defects: A Narrative Review. Cureus.

[29] Shoeibi N., Khazaei S., Motamed Shariati M. (2025). Xerophthalmia and Nyctalopia as Presenting Signs of Vitamin A Deficiency in a Patient with Rapid Intentional Weight Loss: A Case Report and Literature Review. Clin. Case Rep..

[30] Medina-García M., Baeza-Morales A., Martínez-Peinado P., Pascual-García S., Pujalte-Satorre C., Martínez-Espinosa R.M., Sempere-Ortells J.M. (2025). Carotenoids and Their Interaction with the Immune System. Antioxidants.

[31] Fiedor J., Burda K. (2014). Potential Role of Carotenoids as Antioxidants in Human Health and Disease. Nutrients.

[32] Martorell P., Llopis S., Gil J.V., Genovés S., Ramón D., Zacarías L., Rodrigo M.J. (2020). Evaluation of Carotenoids Protection Against Oxidative Stress in the Animal Model *Caenorhabditis elegans*. Methods Mol. Biol..

[33] Yang H., Gu J., Zhu Q., Lu H., Wang K., Ni X., Lu Y., Lu L. (2015). Protection of Acute GVHD by All-Trans Retinoic Acid through Suppression of T Cell Expansion and Induction of Regulatory T Cells through IL-2 Signaling. Int. Immunopharmacol..

[34] Jeong M., Cortopassi F., See J.X., De La Torre C., Cerwenka A., Stojanovic A. (2024). Vitamin A-Treated Natural Killer Cells Reduce Interferon-Gamma Production and Support Regulatory T-Cell Differentiation. Eur. J. Immunol..

[35] Mora J.R., Iwata M., von Andrian U.H. (2008). Vitamin Effects on the Immune System: Vitamins A and D Take Centre Stage. Nat. Rev. Immunol..

[36] Kim Y.K., Giordano E., Hammerling U., Champaneri D., von Lintig J., Hussain M.M., Quadro L. (2025). The Intestine-Specific Homeobox (ISX) Modulates β-Carotene-Dependent Regulation of Microsomal Triglyceride Transfer Protein (MTP) in a Tissue-Specific Manner. Biochim. Biophys. Acta Mol. Cell Biol. Lipids.

[37] Kołodziejczyk A.M., Karwowski B. (2025). Anti-DNA Damage Mechanisms and the Role of Carotenoids, Vitamin A, and Its Derivatives. Nutrients.

[38] Xie G., Cao S., Wang G., Zhang X., Zhang Y., Wu H., Shen S., Le J., Li K., Huang Z. (2025). Vitamin A and Its Influence on Tumour Extracellular Matrix. Discov. Oncol..

[39] Everts H.B., Akuailou E.N. (2021). Retinoids in Cutaneous Squamous Cell Carcinoma. Nutrients.

[40] Karthik N., Sharma S. (2025). Vitamin A Deficiency Masquerading as Cancer-Associated Retinopathy. Can. J. Ophthalmol..

[41] Ocadiz-Delgado R., Serafin-Higuera N., Alvarez-Rios E., García-Villa E., Tinajero-Rodríguez M., Rodríguez-Uribe G., Escobar-Wilches D.C., Estela Albino-Sánchez M., Ramírez-Rosas A., Sierra-Santoyo A. (2021). Vitamin A deficiency in K14E7HPV expressing transgenic mice facilitates the formation of malignant cervical lesions. APMIS.

[42] Damaj N., Elias N., Zeidan T., Kattan J. (2025). Understanding the differentiation syndrome in acute promyelocytic leukemia: A comprehensive updated review. Invest. New Drugs.

[43] Zhang A., Qiu S. (2025). Advances in RARα fusion genes in acute promyelocytic leukemia. Exp. Hematol..

[44] Liu S., Zhan W., He X., Hao M., Shen W., Zhang X., Wang M., Li Z., Hou R., Ou Z. (2024). ATPR induces acute promyelocytic leukemia cells differentiation and cycle arrest via the lncRNA CONCR/DDX11/PML-RARα signaling axis. Gene.

[45] Zhou G.B., Zhang J., Wang Z.Y., Chen S.J., Chen Z. (2007). Treatment of acute promyelocytic leukaemia with all-trans retinoic acid and arsenic trioxide: A paradigm of synergistic molecular targeting therapy. Philos. Trans. R. Soc. Lond. B Biol. Sci..

[46] de Almeida L.Y., Pereira-Martins D.A., Weinhäuser I., Ortiz C., Cândido L.A., Lange A.P., De Abreu N.F., Mendonza S.E.S., de Deus Wagatsuma V.M., Do Nascimento M.C. (2021). The combination of gefitinib with ATRA and ATO induces myeloid differentiation in acute promyelocytic leukemia resistant cells. Front. Oncol..

[47] Li L., Xi H.M., Lu H., Cai X. (2024). Combination of ethacrynic acid and ATRA triggers differentiation and/or apoptosis of acute myeloid leukemia cells through ROS. Anticancer Agents Med. Chem..

[48] Hu L., Li Q., Wang J., Wang H., Ren X., Huang K., Wang Y., Liang X., Pu L., Xiong S. (2024). The CDK4/6 inhibitor palbociclib synergizes with ATRA to induce differentiation in AML. Mol. Cancer Ther..

[49] Xi H.M., Lu H., Weng X.Q., Sheng Y., Wu J., Li L., Cai X. (2023). Combined application of salinomycin and ATRA induces apoptosis and differentiation of acute myeloid leukemia cells by inhibiting WNT/β-catenin pathway. Anticancer Agents Med. Chem..

[50] Parsa L., Motafakkerazad R., Soheyli S.T., Haratian A., Kosari-Nasab M., Mahdavi M. (2023). Silymarin in combination with ATRA enhances apoptosis induction in human acute promyelocytic NB4 cells. Toxicon.

[51] Samarkhazan N.S., Yekta R., Sayadi M., Tackallou S.H., Safaralizadeh R., Mahdavi M. (2020). 2-NDC from dithiocarbamates improves ATRA efficiency and ROS-induced apoptosis via downregulation of Bcl2 and Survivin in human acute promyelocytic NB4 cells. Hum. Exp. Toxicol..

[52] Suzuki S., Liu J., Sato Y., Miyake R., Suzuki S., Okitsu Y., Fukuda T., Isaji T., Gu J., Takahashi S. (2024). Fucosylation inhibitor 6-alkynylfucose enhances the ATRA-induced differentiation effect on acute promyelocytic leukemia cells. Biochem. Biophys. Res. Commun..

[53] Lancet J.E., Moseley A.B., Coutre S.E., DeAngelo D.J., Othus M., Tallman M.S., Litzow M.R., Komrokji R.S., Erba H.P., Appelbaum F.R. (2020). A phase 2 study of ATRA, arsenic trioxide, and gemtuzumab ozogamicin in patients with high-risk APL (SWOG 0535). Blood Adv..

[54] Jen W.Y., Marvin-Peek J., Kantarjian H.M., Alvarado Y., Borthakur G., Jabbour E., Wierda W., Kadia T.M., Daver N.G., DiNardo C.D. (2025). Long-term follow-up of a phase 2 study of all-trans retinoic acid, arsenic trioxide, and gemtuzumab ozogamicin in acute promyelocytic leukemia. Cancer.

[55] Wang H.Y., Gong S., Li G.H., Yao Y.Z., Zheng Y.S., Lu X.H., Wei S.H., Qin W.W., Liu H.B., Wang M.C. (2022). An effective and chemotherapy-free strategy of all-trans retinoic acid and arsenic trioxide for acute promyelocytic leukemia in all risk groups (APL15 trial). Blood Cancer J..

[56] Ramchatesingh B., Martínez Villarreal A., Arcuri D., Lagacé F., Setah S.A., Touma F., Al-Badarin F., Litvinov I.V. (2022). The use of retinoids for the prevention and treatment of skin cancers: An updated review. Int. J. Mol. Sci..

[57] Asgari M.M., Brasky T.M., White E. (2012). Association of vitamin A and carotenoid intake with melanoma risk in a large prospective cohort. J. Investig. Dermatol..

[58] Siddikuzzaman, Grace V.M. (2014). Anti-metastatic study of liposome-encapsulated all-trans retinoic acid (ATRA) in B16F10 melanoma cells-implanted C57BL/6 mice. Cancer Investig..

[59] Melnik B.C. (2017). p53: Key conductor of all anti-acne therapies. J. Transl. Med..

[60] Du Y., Li L.L., Chen H., Wang C., Qian X.W., Feng Y.B., Zhang L., Chen F.H. (2018). A novel all-trans retinoic acid derivative inhibits proliferation and induces apoptosis of myelodysplastic syndromes cell line SKM-1 cells via up-regulating p53. Int. Immunopharmacol..

[61] Chen X., Zhang Z., Hu H., Cai Y., Yang T., Liu O. (2025). All-trans retinoic acid exacerbates Stevens–Johnson syndrome and toxic epidermal necrolysis via TNF signaling pathways: A network toxicology, molecular docking, and experimental study. Toxicol. Appl. Pharmacol..

[62] Ianhez M., Fleury L.F., Miot H.A., Bagatin E. (2013). Retinoids for prevention and treatment of actinic keratosis. An. Bras. Dermatol..

[63] Shalinsky D.R., Bischoff E.D., Gregory M.L., Gottardis M.M., Hayes J.S., Lamph W.W., Heyman R.A., Shirley M.A., Cooke T.A., Davies P.J. (1995). Retinoid-induced suppression of squamous cell differentiation in human oral squamous cell carcinoma xenografts (line 1483) in athymic nude mice. Cancer Res..

[64] Shapiro S.S., Seiberg M., Cole C.A. (2013). Vitamin A and its derivatives in experimental photocarcinogenesis: Preventive effects and relevance to humans. J. Drugs Dermatol..

[65] Kraemer K.H., DiGiovanna J.J., Moshell A.N., Tarone R.E., Peck G.L. (1988). Prevention of skin cancer in xeroderma pigmentosum with the use of oral isotretinoin. N. Engl. J. Med..

[66] Mahamat-Saleh Y., Savoye I., Cervenka I., Al-Rahmoun M., Cadeau C., Boutron-Ruault M.C., Kvaskoff M. (2022). Dietary antioxidant supplements and risk of keratinocyte cancers in women: A prospective cohort study. Eur. J. Nutr..

[67] El Hajj H., Khalil B., Ghandour B., Nasr R., Shahine S., Ghantous A., Abdel-Samad R., Sinjab A., Hasegawa H., Jabbour M. (2014). Preclinical efficacy of the synthetic retinoid ST1926 for treating adult T-cell leukemia/lymphoma. Blood.

[68] Goli M., Sandilya V., Ghandour B., Hajj H.E., Kobeissy F., Darwiche N., Mechref Y. (2025). Exploring the anti-leukemic effect of the synthetic retinoid ST1926 on malignant T cells: A comprehensive proteomics approach. Int. J. Mol. Sci..

[69] Caselli E., Galvan M., Santoni F., Alvarez S., de Lera A.R., Ivanova D., Gronemeyer H., Caruso A., Guidoboni M., Cassai E. (2008). Retinoic acid analogues inhibit human herpesvirus 8 replication. Antivir. Ther..

[70] González de Arriba A., Pérez-Gala S., Goiriz-Valdés R., Ríos-Buceta L., García-Díez A. (2007). Sarcoma de Kaposi clásico tratado con alitretinoína tópica [Kaposi’s sarcoma treated with topical alitretinoin]. Actas Dermosifiliogr..

[71] Oliveira S., Costa J., Faria I., Guerreiro S.G., Fernandes R. (2019). Vitamin A enhances macrophages activity against B16-F10 malignant melanocytes: A new player for cancer immunotherapy?. Medicina.

[72] Jobani B.M., Najafzadeh N., Mazani M., Arzanlou M., Vardin M.M. (2018). Molecular mechanism and cytotoxicity of allicin and all-trans retinoic acid against CD44^+^ versus CD117^+^ melanoma cells. Phytomedicine.

[73] Kanai M., Shinagawa A., Ota M., Virgona N., Yano T. (2024). Resveratrol can differentiate human melanoma stem-like cells from spheroids treated with all-trans retinoic acid. Anticancer Res..

[74] Wang Y.B., Sun B., Han B. (2018). Retinoic acid increases the anticancer effect of paclitaxel by inducing differentiation of cancer stem cells in melanoma. Pharmazie.

[75] Kim G., Bhattarai P.Y., Oh C.H., Choi H.S. (2019). All-trans retinoic acid overcomes acquired resistance to PLX4032 via inhibition of PIN1 in melanoma cells. Anticancer Res..

[76] Grace V.M.B., Saranya S., Wilson D.D. (2021). Protective role of all-trans retinoic acid on B16F10 melanoma cell line metastasis in C57BL/6 mice by enhancing RAR-β protein and homeostasis maintenance. J. Histotechnol..

[77] Yin W., Song Y., Liu Q., Wu Y., He R. (2017). Topical treatment of all-trans retinoic acid inhibits murine melanoma partly by promoting CD8^+^ T-cell immunity. Immunology.

[78] Chen J., Cao X., An Q., Zhang Y., Li K., Yao W., Shi F., Pan Y., Jia Q., Zhou W. (2018). Inhibition of cancer stem cell like cells by a synthetic retinoid. Nat. Commun..

[79] Tobin R.P., Cogswell D.T., Cates V.M., Davis D.M., Borgers J.S.W., Van Gulick R.J., Katsnelson E., Couts K.L., Jordan K.R., Gao D. (2023). Targeting MDSC differentiation using ATRA: A phase I/II clinical trial combining pembrolizumab and all-trans retinoic acid for metastatic melanoma. Clin. Cancer Res..

[80] Tobin R.P., Jordan K.R., Robinson W.A., Davis D., Borges V.F., Gonzalez R., Lewis K.D., McCarter M.D. (2018). Targeting myeloid-derived suppressor cells using all-trans retinoic acid in melanoma patients treated with ipilimumab. Int. Immunopharmacol..

[81] Mittal V., So J.Y., Li S., Swetter S.M., Linos E., Van Horn L., Neuhouser M.L., Stefanick M.L., Tang J.Y. (2024). Associations between dietary and supplemental vitamin A intake and melanoma and non-melanoma skin cancer. Skin Health Dis..

[82] Aborode A.T., Onifade I.A., Olorunshola M.M., Adenikinju G.O., Aruorivwooghene I.J., Femi A.C., Osayawe O.J., Osinuga A., Omojowolo E.A., Adeoye A.F. (2024). Biochemical mechanisms and molecular interactions of vitamins in cancer therapy. Cancer Pathog. Ther..

[83] Manoochehri H., Farrokhnia M., Sheykhhasan M., Mahaki H., Tanzadehpanah H. (2024). Key target genes related to anti-breast cancer activity of ATRA: A network pharmacology, molecular docking, and experimental investigation. Heliyon.

[84] Peng C., Zeleznik O.A., Shutta K.H., Rosner B.A., Kraft P., Clish C.B., Stampfer M.J., Willett W.C., Tamimi R.M., Eliassen A.H. (2022). A metabolomics analysis of circulating carotenoids and breast cancer risk. Cancer Epidemiol. Biomark. Prev..

[85] Dehnavi M.K., Ebrahimpour-Koujan S., Lotfi K., Azadbakht L. (2024). The association between circulating carotenoids and risk of breast cancer: A systematic review and dose-response meta-analysis of prospective studies. Adv. Nutr..

[86] Yan B., Lu M.S., Wang L., Mo X.F., Luo W.P., Du Y.F., Zhang C.X. (2016). Specific serum carotenoids are inversely associated with breast cancer risk among Chinese women: A case–control study. Br. J. Nutr..

[87] Eliassen A.H., Liao X., Rosner B., Tamimi R.M., Tworoger S.S., Hankinson S.E. (2015). Plasma carotenoids and risk of breast cancer over 20 y of follow-up. Am. J. Clin. Nutr..

[88] El Habre R., Aoun R., Tahtouh R., Hilal G. (2024). All-trans-retinoic acid modulates glycolysis via H19 and telomerase: The role of miR-let-7a in estrogen receptor-positive breast cancer cells. BMC Cancer.

[89] Caricasulo M.A., Zanetti A., Terao M., Garattini E., Paroni G. (2024). Cellular and micro-environmental responses influencing the antitumor activity of all-trans retinoic acid in breast cancer. Cell Commun. Signal..

[90] Byun S., Shin S.H., Lee E., Lee J., Lee S.Y., Farrand L., Jung S.K., Cho Y.Y., Um S.J., Sin H.S. (2015). The retinoic acid derivative, ABPN, inhibits pancreatic cancer through induction of Nrdp1. Carcinogenesis.

[91] Chronopoulos A., Robinson B., Sarper M., Cortes E., Auernheimer V., Lachowski D., Attwood S., García R., Ghassemi S., Fabry B. (2016). ATRA mechanically reprograms pancreatic stellate cells to suppress matrix remodelling and inhibit cancer cell invasion. Nat. Commun..

[92] Kuroda H., Tachikawa M., Uchida Y., Inoue K., Ohtsuka H., Ohtsuki S., Unno M., Terasaki T. (2017). All-trans retinoic acid enhances gemcitabine cytotoxicity in human pancreatic cancer cell line AsPC-1 by up-regulating protein expression of deoxycytidine kinase. Eur. J. Pharm. Sci..

[93] Wang K., Baldwin G.S., Nikfarjam M., He H. (2019). Antitumor effects of all-trans retinoic acid and its synergism with gemcitabine are associated with downregulation of p21-activated kinases in pancreatic cancer. Am. J. Physiol. Gastrointest. Liver Physiol..

[94] Zhang T., Chen H., Qin S., Wang M., Wang X., Zhang X., Liu F., Zhang S. (2016). The association between dietary vitamin A intake and pancreatic cancer risk: A meta-analysis of 11 studies. Biosci. Rep..

[95] Huang X., Gao Y., Zhi X., Ta N., Jiang H., Zheng J. (2016). Association between vitamin A, retinol and carotenoid intake and pancreatic cancer risk: Evidence from epidemiologic studies. Sci. Rep..

[96] Kocher H.M., Basu B., Froeling F.E.M., Sarker D., Slater S., Carlin D., deSouza N.M., De Paepe K.N., Goulart M.R., Hughes C. (2020). Phase I clinical trial repurposing all-trans retinoic acid as a stromal targeting agent for pancreatic cancer. Nat. Commun..

[97] Xue Y., Harris E., Wang W., Baybutt R.C. (2015). Vitamin A depletion induced by cigarette smoke is associated with an increase in lung cancer-related markers in rats. J. Biomed Sci..

[98] Okayasu I., Hana K., Nemoto N., Yoshida T., Saegusa M., Yokota-Nakatsuma A., Song S.Y., Iwata M. (2016). Vitamin A inhibits development of dextran sulfate sodium-induced colitis and colon cancer in a mouse model. Biomed Res. Int..

[99] Luo P., Zheng L., Zou J., Chen T., Zou J., Li W., Chen Q., Qian B. (2023). Insights into vitamin A in bladder cancer, lack of attention to gut microbiota?. Front. Immunol..

[100] Tratnjek L., Jeruc J., Romih R., Zupančič D. (2021). Vitamin A and retinoids in bladder cancer chemoprevention and treatment: A narrative review of current evidence, challenges and future prospects. Int. J. Mol. Sci..

[101] Mere Del Aguila E., Tang X.H., Gudas L.J. (2022). Pancreatic ductal adenocarcinoma: New insights into the actions of vitamin A. Oncol. Res. Treat..

[102] Hrabak P., Zelenkova M., Krechler T., Soupal J., Vocka M., Hanus T., Petruzelka L., Svacina S., Zak A., Zima T. (2024). Levels of retinol and retinoic acid in pancreatic cancer, type-2 diabetes and chronic pancreatitis. Biomed Pap. Med. Fac. Univ. Palacky Olomouc Czech Repub..

[103] Bazhin A.V., Bleul T., de Lera A.R., Werner J., Rühl R. (2016). Relationship between all-trans-13,14-dihydro retinoic acid and pancreatic adenocarcinoma. Pancreas.

[104] Groener J.B., Gelen D., Mogler C., Herpel E., Toth C., Kender Z., Peichl M., Haufe S., Haberkorn U., Sulaj A. (2019). BRAF V600E and retinoic acid in radioiodine-refractory papillary thyroid cancer. Horm. Metab. Res..

[105] Fu M., Zhang Y., Peng B., Luo N., Zhang Y., Zhu W., Yang F., Chen Z., Zhang Q., Li Q. (2024). All-trans retinoic acid inhibits glioblastoma progression and attenuates radiation-induced brain injury. JCI Insight.

[106] Jones T., Zhang B., Major S., Webb A. (2020). All-trans retinoic acid eluting poly(diol citrate) wafers for treatment of glioblastoma. J. Biomed Mater. Res. B Appl. Biomater..

[107] Ye L., Zhang L., Li R., Pan X., Li J., Dou S., Jiang W., Wang C., Chen W., Zhu G. (2023). Combined all-trans retinoic acid with low-dose apatinib in treatment of recurrent/metastatic head and neck adenoid cystic carcinoma: A single-center, secondary analysis of a phase II study. Cancer Med..

[108] Işlek Köklü Z., Şanverdi E.L., Karadağ B., Üçişik M.H., Taşkan E., Şahin F. (2024). Combinational therapy of all-trans retinoic acid (ATRA) and sphingomyelin induces apoptosis and cell cycle arrest in B16F10 melanoma cancer cells. Turk. J. Biol..

[109] Younes M., Loubnane G., Sleiman C., Rizk S. (2024). Tocotrienol isoforms: The molecular mechanisms underlying their effects in cancer therapy and their implementation in clinical trials. J. Integr. Med..

[110] Chiaramonte R., Sauro G., Giannandrea D., Limonta P., Casati L. (2025). Molecular insights in the anticancer activity of natural tocotrienols: Targeting mitochondrial metabolism and cellular redox homeostasis. Antioxidants.

[111] Kaye A.D., Thomassen A.S., Mashaw S.A., MacDonald E.M., Waguespack A., Hickey L., Singh A., Gungor D., Kallurkar A., Kaye A.M. (2025). Vitamin E (α-tocopherol): Emerging clinical role and adverse risks of supplementation in adults. Cureus.

[112] Es-Sai B., Wahnou H., Benayad S., Rabbaa S., Laaziouez Y., El Kebbaj R., Limami Y., Duval R.E. (2025). Gamma-tocopherol: A comprehensive review of its antioxidant, anti-inflammatory, and anticancer properties. Molecules.

[113] Pierpaoli E., Viola V., Pilolli F., Piroddi M., Galli F., Provinciali M. (2010). Gamma- and delta-tocotrienols exert a more potent anticancer effect than alpha-tocopheryl succinate on breast cancer cell lines irrespective of HER-2/neu expression. Life Sci..

[114] Jiang Q. (2017). Natural forms of vitamin E as effective agents for cancer prevention and therapy. Adv. Nutr..

[115] Sailo B.L., Banik K., Padmavathi G., Javadi M., Bordoloi D., Kunnumakkara A.B. (2018). Tocotrienols: The promising analogues of vitamin E for cancer therapeutics. Pharmacol. Res..

[116] Khalid A.Q., Bhuvanendran S., Magalingam K.B., Ramdas P., Radhakrishnan A.K. (2025). Inhibition of proliferation and induction of apoptosis by gamma- or delta-tocotrienols in human colorectal carcinoma cells. Biomed Res. Int..

[117] Jang Y., Park N.Y., Rostgaard-Hansen A.L., Huang J., Jiang Q. (2016). Vitamin E metabolite 13′-carboxychromanols inhibit pro-inflammatory enzymes, induce apoptosis and autophagy in human cancer cells by modulating sphingolipids and suppress colon tumor development in mice. Free Radic. Biol. Med..

[118] Guan F., Li G., Liu A.B., Lee M.J., Yang Z., Chen Y.K., Lin Y., Shih W., Yang C.S. (2012). δ- and γ-tocopherols, but not α-tocopherol, inhibit colon carcinogenesis in azoxymethane-treated F344 rats. Cancer Prev. Res..

[119] Falsetti I., Palmini G., Zonefrati R., Vasa K., Donati S., Aurilia C., Baroncelli A., Viglianisi C., Ranaldi F., Iantomasi T. (2025). Antiproliferative role of natural and semi-synthetic tocopherols on colorectal cancer cells overexpressing the estrogen receptor β. Int. J. Mol. Sci..

[120] Khalid A.Q., Zaidan T.N., Bhuvanendran S., Magalingam K.B., Mohamedahmed S.M., Ramdas P., Radhakrishnan A.K. (2025). Insights into the anticancer mechanisms modulated by gamma and delta tocotrienols in colorectal cancers. Nutr. Rev..

[121] Husain K., Coppola D., Yang C.S., Malafa M.P. (2024). Effect of vitamin E δ-tocotrienol and aspirin on Wnt signaling in human colon cancer stem cells and in adenoma development in APCmin/+ mice. Carcinogenesis.

[122] Schlörmann W., Liao S., Dinc T., Lorkowski S., Wallert M., Glei M. (2024). Chemopreventive effects of α-tocopherol and its long-chain metabolites α-13′-hydroxy- and α-13′-carboxychromanol in LT97 colon adenoma cells. Food Funct..

[123] Liu K.Y., Wang Q., Nakatsu C.H., Jones-Hall Y., Jiang Q. (2023). Combining gamma-tocopherol and aspirin synergistically suppresses colitis-associated colon tumorigenesis and modulates the gut microbiota in mice, and inhibits the growth of human colon cancer cells. Eur. J. Pharmacol..

[124] Yang C., Zhao Y., Im S., Nakatsu C., Jones-Hall Y., Jiang Q. (2021). Vitamin E delta-tocotrienol and metabolite 13′-carboxychromanol inhibit colitis-associated colon tumorigenesis and modulate gut microbiota in mice. J. Nutr. Biochem..

[125] Bazzaz R., Bijanpour H., Pirouzpanah S.M.B., Yaghmaei P., Rashtchizadeh N. (2019). Adjuvant therapy with γ-tocopherol induces apoptosis in HT-29 colon cancer via cyclin-dependent cell cycle arrest mechanism. J. Biochem. Mol. Toxicol..

[126] Chen J.X., Liu A., Lee M.J., Wang H., Yu S., Chi E., Reuhl K., Suh N., Yang C.S. (2017). δ- and γ-tocopherols inhibit PhIP/DSS-induced colon carcinogenesis by protection against early cellular and DNA damages. Mol. Carcinog..

[127] Yoon H.S., Wu J., Shidal C., Sun Y., Franke A.A., Yang J.J., Braithwaite D., Courtney R., Cai H., Blot W.J. (2024). Associations between plasma tocopherols and lung cancer risk: Results from the Southern Community Cohort Study. Cancer Epidemiol. Biomark. Prev..

[128] Huang J., Weinstein S.J., Yu K., Männistö S., Albanes D. (2020). A prospective study of serum vitamin E and 28-year risk of lung cancer. J. Natl. Cancer Inst..

[129] Zhu Y.J., Bo Y.C., Liu X.X., Qiu C.G. (2017). Association of dietary vitamin E intake with risk of lung cancer: A dose-response meta-analysis. Asia Pac. J. Clin. Nutr..

[130] Wiel C., Le Gal K., Ibrahim M.X., Jahangir C.A., Kashif M., Yao H., Ziegler D.V., Xu X., Ghosh T., Mondal T. (2019). BACH1 stabilization by antioxidants stimulates lung cancer metastasis. Cell.

[131] Rajasinghe L.D., Hutchings M., Gupta S.V. (2019). Delta-tocotrienol modulates glutamine dependence by inhibiting ASCT2 and LAT1 transporters in non-small cell lung cancer (NSCLC) cells: A metabolomic approach. Metabolites.

[132] Rajasinghe L.D., Pindiprolu R.H., Gupta S.V. (2018). Delta-tocotrienol inhibits non-small-cell lung cancer cell invasion via the inhibition of NF-κB, uPA activator, and MMP-9. Onco Targets Ther..

[133] Uchihara Y., Kidokoro T., Tago K., Mashino T., Tamura H., Funakoshi-Tago M. (2018). A major component of vitamin E, α-tocopherol, inhibits the anti-tumor activity of crizotinib against cells transformed by EML4-ALK. Eur. J. Pharmacol..

[134] Daifuku R., Koratich M., Stackhouse M. (2018). Vitamin E phosphate nucleoside prodrugs: A platform for intracellular delivery of monophosphorylated nucleosides. Pharmaceuticals.

[135] Mondul A.M., Moore S.C., Weinstein S.J., Karoly E.D., Sampson J.N., Albanes D. (2015). Metabolomic analysis of prostate cancer risk in a prospective cohort: The alpha-tocopherol, beta-carotene cancer prevention (ATBC) study. Int. J. Cancer.

[136] Antwi S.O., Steck S.E., Zhang H., Stumm L., Zhang J., Hurley T.G., Hebert J.R. (2015). Plasma carotenoids and tocopherols in relation to prostate-specific antigen (PSA) levels among men with biochemical recurrence of prostate cancer. Cancer Epidemiol..

[137] Wang H., Hong J., Yang C.S. (2016). δ-Tocopherol inhibits receptor tyrosine kinase-induced AKT activation in prostate cancer cells. Mol. Carcinog..

[138] Yeganehjoo H., DeBose-Boyd R., McFarlin B.K., Mo H. (2017). Synergistic impact of d-δ-tocotrienol and geranylgeraniol on the growth and HMG-CoA reductase of human DU145 prostate carcinoma cells. Nutr. Cancer.

[139] Fajardo A.M., MacKenzie D.A., Olguin S.L., Scariano J.K., Rabinowitz I., Thompson T.A. (2016). Antioxidants abrogate alpha-tocopherylquinone-mediated down-regulation of the androgen receptor in androgen-responsive prostate cancer cells. PLoS ONE.

[140] Wang H., Yang X., Liu A., Wang G., Bosland M.C., Yang C.S. (2018). δ-Tocopherol inhibits the development of prostate adenocarcinoma in prostate-specific Pten-/- mice. Carcinogenesis.

[141] Wang H., Yan W., Sun Y., Yang C.S. (2022). δ-Tocotrienol is the most potent vitamin E form in inhibiting prostate cancer cell growth and inhibits prostate carcinogenesis in Ptenp-/- mice. Cancer Prev. Res..

[142] Sato C., Kaneko S., Sato A., Virgona N., Namiki K., Yano T. (2017). Combination effect of δ-tocotrienol and γ-tocopherol on prostate cancer cell growth. J. Nutr. Sci. Vitaminol..

[143] Fontana F., Moretti R.M., Raimondi M., Marzagalli M., Beretta G., Procacci P., Sartori P., Montagnani Marelli M., Limonta P. (2019). δ-Tocotrienol induces apoptosis, involving endoplasmic reticulum stress and autophagy, and paraptosis in prostate cancer cells. Cell Prolif..

[144] Moore C., Palau V.E., Mahboob R., Lightner J., Stone W., Krishnan K. (2020). Upregulation of pERK and c-JUN by γ-tocotrienol and not α-tocopherol are essential to the differential effect on apoptosis in prostate cancer cells. BMC Cancer.

[145] Tang K.D., Liu J., Russell P.J., Clements J.A., Ling M.T. (2019). Gamma-tocotrienol induces apoptosis in prostate cancer cells by targeting the Ang-1/Tie-2 signalling pathway. Int. J. Mol. Sci..

[146] Huang Y., Wu R., Su Z.Y., Guo Y., Zheng X., Yang C.S., Kong A.N. (2017). A naturally occurring mixture of tocotrienols inhibits the growth of human prostate tumor, associated with epigenetic modifications of cyclin-dependent kinase inhibitors p21 and p27. J. Nutr. Biochem..

[147] Sun Z., Ma X., Li J., Fan L., Zhao C., Yin S., Hu H. (2025). δ-Tocotrienol potentiates breast and prostate cancer cells to paclitaxel via suppressing PD-L1-mediated cancer-promoting signaling. Chem. Biol. Drug Des..

[148] Helzlsouer K.J., Huang H.Y., Alberg A.J., Hoffman S., Burke A., Norkus E.P., Morris J.S., Comstock G.W. (2000). Association between alpha-tocopherol, gamma-tocopherol, selenium, and subsequent prostate cancer. J. Natl. Cancer Inst..

[149] Lippman S.M., Klein E.A., Goodman P.J., Lucia M.S., Thompson I.M., Ford L.G., Parnes H.L., Minasian L.M., Gaziano J.M., Hartline J.A. (2009). Effect of selenium and vitamin E on risk of prostate cancer and other cancers: The Selenium and Vitamin E Cancer Prevention Trial (SELECT). JAMA.

[150] Klein E.A., Thompson I.M., Tangen C.M., Crowley J.J., Lucia M.S., Goodman P.J., Minasian L.M., Ford L.G., Parnes H.L., Gaziano J.M. (2011). Vitamin E and the risk of prostate cancer: The Selenium and Vitamin E Cancer Prevention Trial (SELECT). JAMA.

[151] Drotleff A.M., Büsing A., Willenberg I., Empl M.T., Steinberg P., Ternes W. (2015). HPLC separation of vitamin E and its oxidation products and effects of oxidized tocotrienols on the viability of MCF-7 breast cancer cells in vitro. J. Agric. Food Chem..

[152] Alawin O.A., Ahmed R.A., Ibrahim B.A., Briski K.P., Sylvester P.W. (2016). Antiproliferative effects of γ-tocotrienol are associated with lipid raft disruption in HER2-positive human breast cancer cells. J. Nutr. Biochem..

[153] Ahmed R.A., Alawin O.A., Sylvester P.W. (2016). γ-Tocotrienol reversal of epithelial-to-mesenchymal transition in human breast cancer cells is associated with inhibition of canonical Wnt signalling. Cell Prolif..

[154] Diao Q.X., Zhang J.Z., Zhao T., Xue F., Gao F., Ma S.M., Wang Y. (2016). Vitamin E promotes breast cancer cell proliferation by reducing ROS production and p53 expression. Eur. Rev. Med. Pharmacol. Sci..

[155] Ding Y., Fan J., Fan Z., Zhang K. (2021). γ-Tocotrienol reverses multidrug resistance of breast cancer cells through the regulation of the γ-tocotrienol–NF-κB–P-gp axis. J. Steroid Biochem. Mol. Biol..

[156] Ding Y., Peng Y., Deng L., Fan J., Huang B. (2017). Gamma-tocotrienol reverses multidrug resistance of breast cancer cells with a mechanism distinct from that of atorvastatin. J. Steroid Biochem. Mol. Biol..

[157] Bak M.J., Das Gupta S., Wahler J., Lee H.J., Li X., Lee M.J., Yang C.S., Suh N. (2017). Inhibitory effects of γ- and δ-tocopherols on estrogen-stimulated breast cancer in vitro and in vivo. Cancer Prev. Res..

[158] Ye J., Dong W., Yang Y., Hao H., Liao H., Wang B., Han X., Jin Y., Xia X., Liu Y. (2017). Vitamin E-rich nanoemulsion enhances the antitumor efficacy of low-dose paclitaxel by driving Th1 immune response. Pharm. Res..

[159] Sailo B.L., Chauhan S., Hegde M., Girisa S., Alqahtani M.S., Abbas M., Goel A., Sethi G., Kunnumakkara A.B. (2025). Therapeutic potential of tocotrienols as chemosensitizers in cancer therapy. Phytother. Res..

[160] Jiang W., Fan Q., Wang J., Zhang B., Hao T., Chen Q., Li L., Chen L., Cui H., Li Z. (2021). PEGylated phospholipid micelles containing D-α-tocopheryl succinate as multifunctional nanocarriers for enhancing the antitumor efficacy of doxorubicin. Int. J. Pharm..

[161] Opoku-Damoah Y., Zhang R., Ta H.T., Xu Z.P. (2021). Vitamin E-facilitated carbon monoxide pro-drug nanomedicine for efficient light-responsive combination cancer therapy. Biomater. Sci..

[162] Queiroz Schmidt F.M., Serna González C.V., Mattar R.C., Lopes L.B., Santos M.F., Santos V.L.C.G. (2022). Topical application of a cream containing nanoparticles with vitamin E for radiodermatitis prevention in women with breast cancer: A randomized, triple-blind, controlled pilot trial. Eur. J. Oncol. Nurs..

[163] Long X., Guo J., Yin Y., Cheng M., Zhang X., Zhang J., Wang P., Zang J., Zhao L. (2023). A blinded-endpoint, randomized controlled trial of Sanyrene with natural active ingredient for prophylaxis of radiation dermatitis in patients receiving radiotherapy. Radiat. Oncol..

[164] Moustafa I., Connolly C., Anis M., Mustafa H., Oosthuizen F., Viljoen M. (2024). A prospective study to evaluate the efficacy and safety of vitamin E and levocarnitine prophylaxis against doxorubicin-induced cardiotoxicity in adult breast cancer patients. J. Oncol. Pharm. Pract..

[165] Kjær I.M., Kahns S., Timm S., Andersen R.F., Madsen J.S., Jakobsen E.H., Tabor T.P., Jakobsen A., Bechmann T. (2023). Phase II trial of delta-tocotrienol in neoadjuvant breast cancer with evaluation of treatment response using ctDNA. Sci. Rep..

[166] Husain K., Centeno B.A., Coppola D., Trevino J., Sebti S.M., Malafa M.P. (2017). δ-Tocotrienol, a natural form of vitamin E, inhibits pancreatic cancer stem-like cells and prevents pancreatic cancer metastasis. Oncotarget.

[167] Palau V.E., Chakraborty K., Wann D., Lightner J., Hilton K., Brannon M., Stone W., Krishnan K. (2018). γ-Tocotrienol induces apoptosis in pancreatic cancer cells by upregulation of ceramide synthesis and modulation of sphingolipid transport. BMC Cancer.

[168] Francois R.A., Zhang A., Husain K., Wang C., Hutchinson S., Kongnyuy M., Batra S.K., Coppola D., Sebti S.M., Malafa M.P. (2019). Vitamin E δ-tocotrienol sensitizes human pancreatic cancer cells to TRAIL-induced apoptosis through proteasome-mediated down-regulation of c-FLIPs. Cancer Cell Int..

[169] Tang S., Kapoor E., Ding L., Yu A., Tang W., Hang Y., Smith L.M., Sil D., Oupický D. (2023). Effect of tocopherol conjugation on polycation-mediated siRNA delivery to orthotopic pancreatic tumors. Biomater. Adv..

[170] Behera C., Kaur Sandha K., Banjare N., Kumar Shukla M., Mudassir Ali S., Singh M., Gupta P.N. (2024). Biodegradable nanocarrier of gemcitabine and tocopherol succinate synergistically ameliorates anti-proliferative response in MIA PaCa-2 cells. Int. J. Pharm..

[171] Pereira-Silva M., Miranda-Pastoriza D., Diaz-Gomez L., Sotelo E., Paiva-Santos A.C., Veiga F., Concheiro A., Alvarez-Lorenzo C. (2024). Gemcitabine–vitamin E prodrug-loaded micelles for pancreatic cancer therapy. Pharmaceutics.

[172] Pereira-Silva M., Diaz-Gomez L., Blanco-Fernandez B., Paiva-Santos A.C., Veiga F., Concheiro A., Alvarez-Lorenzo C. (2025). Biomimetic cancer cell membrane-enriched vitamin E-stapled gemcitabine-loaded TPGS micelles for pancreatic cancer therapy. Drug Deliv..

[173] Springett G.M., Husain K., Neuger A., Centeno B., Chen D.T., Hutchinson T.Z., Lush R.M., Sebti S., Malafa M.P. (2015). A phase I safety, pharmacokinetic, and pharmacodynamic presurgical trial of vitamin E δ-tocotrienol in patients with pancreatic ductal neoplasia. EBioMedicine.

[174] Mahipal A., Klapman J., Vignesh S., Yang C.S., Neuger A., Chen D.T., Malafa M.P. (2016). Pharmacokinetics and safety of vitamin E δ-tocotrienol after single and multiple doses in healthy subjects with measurement of vitamin E metabolites. Cancer Chemother. Pharmacol..

[175] Li D., Tang H., Wei P., Zheng J., Daniel C.R., Hassan M.M. (2019). Vitamin C and vitamin E mitigate the risk of pancreatic ductal adenocarcinoma from meat-derived mutagen exposure in adults in a case-control study. J. Nutr..

[176] Zhao M., Ye M., Zhao Y. (2025). Causal link between dietary antioxidant vitamins intake, oxidative stress injury biomarkers and colorectal cancer: A Mendelian randomization study. Medicine.

[177] Raunkilde L., Hansen T.F., Havelund B.M., Thomsen C.B., Rafaelsen S.R., Lindebjerg J., Jensen L.H. (2023). Delta tocotrienol as a supplement to FOLFOXIRI in first-line treatment of metastatic colorectal cancer: A randomized, double-blind, placebo-controlled phase II study. Acta Oncol..

[178] Oliveira L.M., Teixeira F.M.E., Sato M.N. (2018). Impact of retinoic acid on immune cells and inflammatory diseases. Mediators Inflamm..

[179] Snyder L.M., Arora J., Kennett M.J., Weaver V., Cantorna M.T. (2020). Retinoid signaling in intestinal epithelial cells is essential for early survival from gastrointestinal infection. Front. Immunol..

[180] Lavudi K., Nuguri S.M., Olverson Z., Dhanabalan A.K., Patnaik S., Kokkanti R.R. (2023). Targeting the retinoic acid signaling pathway as a modern precision therapy against cancers. Front. Cell Dev. Biol..

[181] Nagai Y., Ambinder A.J. (2023). The promise of retinoids in the treatment of cancer: Neither burnt out nor fading away. Cancers.

[182] Chen Y., Tong X., Lu R., Zhang Z., Ma T. (2024). All-trans retinoic acid in hematologic disorders: Not just acute promyelocytic leukemia. Front. Pharmacol..

[183] Amimo J.O., Michael H., Chepngeno J., Raev S.A., Saif L.J., Vlasova A.N. (2022). Immune impairment associated with vitamin A deficiency: Insights from clinical studies and animal model research. Nutrients.

[184] Bastos Maia S., Rolland Souza A.S., Costa Caminha M.F., Lins da Silva S., Callou Cruz R.S.B.L., Carvalho Dos Santos C., Batista Filho M. (2019). Vitamin A and pregnancy: A narrative review. Nutrients.

[185] Esposito M., Amory J.K., Kang Y. (2024). The pathogenic role of retinoid nuclear receptor signaling in cancer and metabolic syndromes. J. Exp. Med..

[186] Ranasinghe R., Mathai M., Zulli A. (2022). Revisiting the therapeutic potential of tocotrienol. Biofactors.

[187] Jiang Q., Christen S., Shigenaga M.K., Ames B.N. (2001). Gamma-tocopherol, the major form of vitamin E in the US diet, deserves more attention. Am. J. Clin. Nutr..

[188] Niki E. (2021). Lipid oxidation that is, and is not, inhibited by vitamin E: Consideration about physiological functions of vitamin E. Free Radic. Biol. Med..

[189] Moses G. (2021). The safety of commonly used vitamins and minerals. Aust Prescr..

[190] Cammisotto V., Nocella C., Bartimoccia S., Sanguigni V., Francomano D., Sciarretta S., Pastori D., Peruzzi M., Cavarretta E., D’Amico A. (2021). The Role of Antioxidants Supplementation in Clinical Practice: Focus on Cardiovascular Risk Factors. Antioxidants.

